# Nitazoxanide Stimulates Autophagy and Inhibits mTORC1 Signaling and Intracellular Proliferation of *Mycobacterium tuberculosis*

**DOI:** 10.1371/journal.ppat.1002691

**Published:** 2012-05-10

**Authors:** Karen K. Y. Lam, Xingji Zheng, Roberto Forestieri, Aruna D. Balgi, Matt Nodwell, Sarah Vollett, Hilary J. Anderson, Raymond J. Andersen, Yossef Av-Gay, Michel Roberge

**Affiliations:** 1 Department of Biochemistry and Molecular Biology, University of British Columbia, Vancouver, British Columbia, Canada; 2 Division of Infectious Diseases, Department of Medicine, University of British Columbia, Vancouver, British Columbia, Canada; 3 Departments of Chemistry and Earth and Ocean Sciences, University of British Columbia, Vancouver, British Columbia, Canada; University of New Mexico, United States of America

## Abstract

Tuberculosis, caused by *Mycobacterium tuberculosis* infection, is a major cause of morbidity and mortality in the world today. *M. tuberculosis* hijacks the phagosome-lysosome trafficking pathway to escape clearance from infected macrophages. There is increasing evidence that manipulation of autophagy, a regulated catabolic trafficking pathway, can enhance killing of *M. tuberculosis*. Therefore, pharmacological agents that induce autophagy could be important in combating tuberculosis. We report that the antiprotozoal drug nitazoxanide and its active metabolite tizoxanide strongly stimulate autophagy and inhibit signaling by mTORC1, a major negative regulator of autophagy. Analysis of 16 nitazoxanide analogues reveals similar strict structural requirements for activity in autophagosome induction, EGFP-LC3 processing and mTORC1 inhibition. Nitazoxanide can inhibit *M. tuberculosis* proliferation *in vitro*. Here we show that it inhibits *M. tuberculosis* proliferation more potently in infected human THP-1 cells and peripheral monocytes. We identify the human quinone oxidoreductase NQO1 as a nitazoxanide target and propose, based on experiments with cells expressing NQO1 or not, that NQO1 inhibition is partly responsible for mTORC1 inhibition and enhanced autophagy. The dual action of nitazoxanide on both the bacterium and the host cell response to infection may lead to improved tuberculosis treatment.

## Author Summary

Tuberculosis is responsible for approximately 2 million deaths worldwide each year. Current treatment regimens require administration of multiple drugs over several months and resistance to these drugs is on the rise. *Mycobacterium tuberculosis*, the causative agent of the disease, can proliferate within host cells. It has been recently observed that autophagy (cellular self-eating) can kill intracellular *M. tuberculosis*. We report that the antiprotozoal drug nitazoxanide and its metabolite tizoxanide induce autophagy, inhibit signaling by mTORC1, a major negative regulator of autophagy, and prevent *M. tuberculosis* proliferation in infected macrophages. We show that nitazoxanide exerts at least some of its pharmacological effects by targeting the quinone reductase NQO1. Our results uncover a novel mechanism of action for the drug nitazoxanide, and show that pharmacological modulation of autophagy can suppress intracellular *M. tuberculosis* proliferation.

## Introduction

*Mycobacterium tuberculosis* (Mtb) is the bacterial pathogen that causes tuberculosis, a major infectious disease responsible for approximately 2 million deaths worldwide each year [1]. There is a major need for more effective therapy against tuberculosis [2], [3]. Mtb is a highly persistent and successful pathogen in part because of its ability to manipulate intracellular membrane trafficking events in host macrophages [4], [5]. Upon entering the host cell, Mtb resides in single-membraned phagosomes and initiates mechanisms to avoid the innate immune response that can activate macrophages [6]–[9]. A series of fusion events with various endocytic organelles, culminating in fusion with lysosomes, normally converts the phagosome into a phagolysosome that can destroy its microbial contents [7]. Mtb prevents this conversion at an early stage by secreting a protein phosphatase, PtpA, that blocks the acquisition of the vacuolar-type H^+^-ATPase required for acidification of the lumen [10]–[13], limiting the acquisition of lysosomal hydrolases and depleting the phagosome of phosphatidylinositol 3-phosphate [7], [14], [15].

Autophagy is another intracellular membrane trafficking pathway that can play a role in controlling bacterial infection [16], [17]. In this process, cytoplasmic constituents are sequestered in double-membraned structures called autophagosomes that are subsequently targeted for fusion with lysosomes and are degraded [18]. Under basal conditions this degradative pathway is important for recycling intracellular material and organelles to maintain cellular homeostasis. Experimental induction of autophagy in macrophages by starvation, rapamycin, interferon-γ or its downstream effector LRG-47, toll-like receptor stimulation, ATP stimulation, or by small molecules reduced survival of intracellular Mtb [8], [19]–[23]. This was associated with increased acidification of phagosomes and increased colocalization of lysosomal and autophagosomal markers with Mtb-containing phagosomes [8], [19], [20], suggesting the block to phagosome maturation was overcome and fusion with lysosomal and autophagosomal compartments occurred. Further work has shown that the reduced Mtb survival is associated with delivery to the Mtb compartment of autophagosomal protein cargo that is proteolysed to generate cationic peptides that are toxic to Mtb [24], [25].

Autophagy is in part regulated by the mammalian target of rapamycin complex 1 (mTORC1), a nutrient-, energy- and growth factor-sensing master regulator of cell growth and metabolism [26]. mTORC1 is stimulated by growth factors and nutrients to promote anabolic processes such as translation and protein synthesis. Conversely, nutrient deprivation, cellular stress and the chemical rapamycin inhibit mTORC1, leading to the attenuation of anabolic reactions and the induction of autophagic catabolism as a protective function [27].

The evidence supporting a protective, cell-clearing function for autophagy in Mtb-infected macrophages suggests autophagy and mTORC1 signaling as attractive targets for new treatments for tuberculosis. Few studies have explored the use of approved drugs to manipulate autophagy or mTORC1 to combat Mtb infection. We recently reported results of a screen for chemicals that increase autophagosome formation and identified niclosamide, an approved salicylanilide antihelmintic drug, as a potent stimulator of autophagy and inhibitor of mTORC1 signaling [28]. Although niclosamide is very effective in the intestinal tract, it is not a good candidate for Mtb treatment because of its poor absorption. In the present paper we examine whether nitazoxanide (NTZ, 2-acetyloxy-*N*-(5-nitro-2-thiazolyl)benzamide, Alinia), a newer antiparasitic drug that was synthesized based on the structure of niclosamide [29] and that shows good gastrointestinal absorption, might also affect autophagy and mTORC1 activity and be a better drug candidate for Mtb treatment.

We report that NTZ stimulates autophagy, inhibits mTORC1 signaling, and inhibits Mtb intracellular proliferation at concentrations found in the blood in humans after administration of a standard oraldose. We show that NTZ inhibits the enzymatic activity of human quinone oxidoreductase NQO1,probably acting upstream of mTORC1 to stimulate autophagy. We also provide insights into the structural requirements of NTZ for activity. Our work further supports a mechanistic link between autophagy and Mtb proliferation, and presents an additional option for the manipulation of autophagy in the treatment of Mtb.

## Results

### Nitazoxanide modulates autophagy

**Figure 1:**
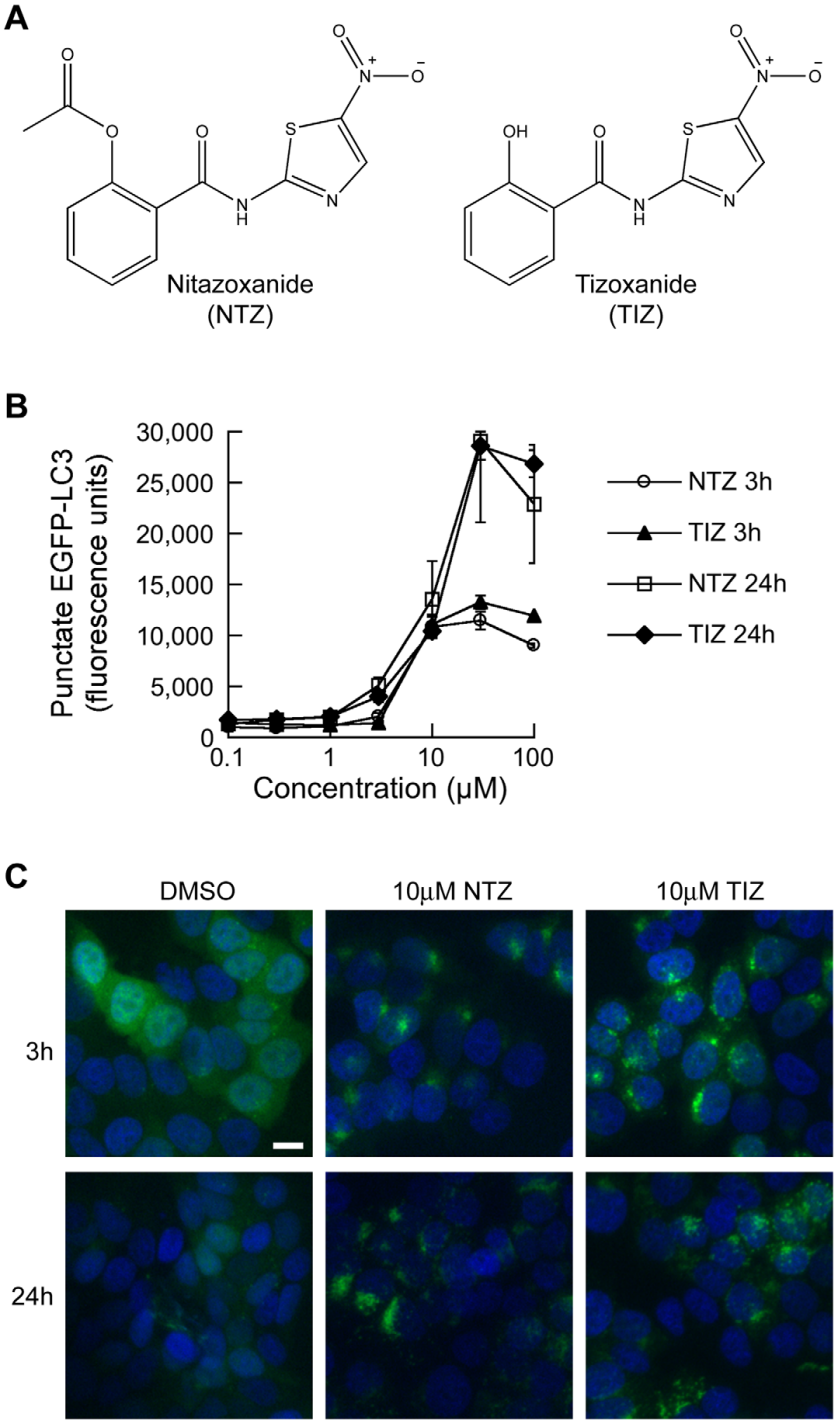
Induction of autophagosomes by NTZ and TIZ. (A)Structures of NTZ and TIZ. (B) Punctate EGFP-LC3 levels in MCF-7 cells stably expressing EGFP-LC3. Accumulation was measured quantitatively by automated microscopy in cells incubated for 3 h or 24 h with different concentrations of NTZ or TIZ. (C) Representative images of cells showing EGFP-LC3 (green) and DNA (blue).Scale bar, 10 µm.

NTZ (Figure 1A) structurally resembles niclosamide, a drug that stimulates autophagy and inhibits signaling by mTORC1, a negative regulator of autophagy [28]. NTZ is a prodrug that is rapidly hydrolyzed in plasma to the active metabolite tizoxanide (TIZ; Figure 1A) [30]. We therefore examined the effects of both NTZ and TIZ on autophagy. The formation of autophagosomes entails the recruitment of cytosolic Atg8/LC3 to nascent autophagosomes [31]. This process can be monitored quantitatively by automated fluorescence microscopy in MCF-7 cells expressing LC3 fused to EGFP (EGFP-LC3) as a shift from diffuse to punctate cytoplasmic fluorescence [28]. Cells were exposed for 3 h or 24 h to concentrations of NTZ or TIZ ranging from 0.1 to 100 µM. Untreated cells showed mostly diffuse cytosolic EGFP-LC3 staining and a low level of punctate EGFP-LC3, representing basal levels of autophagy (Figure 1B, C). After 3 hincubation, NTZ and TIZ caused a concentration-dependent increase in autophagosomes that was detectable at 3 µM and maximal at 30 µM (Figure 1B and Figure S1). After 24 h incubation, autophagosome accumulation was even more pronounced (Figure 1B and Figure S1).

Autophagosome accumulation observed in a static image may reflect increased autophagic flux but has also been observed in the presence of chemicals that decrease autophagic flux by inhibiting the final stage of autophagy in which autophagosomes fuse with lysosomes and are degraded [32]. To distinguish between these possibilities, the effect of NTZ and TIZ on EGFP-LC3 processing and degradation was examined by immunoblotting. Recruitment of LC3 or EGFP-LC3 to autophagosomes involves its proteolytic cleavage and lipidation, yielding a species with increased electrophoretic mobility termed LC3II/EGFP-LC3II [31]. Upon fusion with lysosomes, the contents of autophagosomes, including sequestered LC3, are degraded by lysosomal hydrolases. However, the free EGFP portion is degraded more slowly than LC3 itself, leading to transient accumulation of a band corresponding to the size of EGFP (∼25 kD) [32].

**Figure 2:**
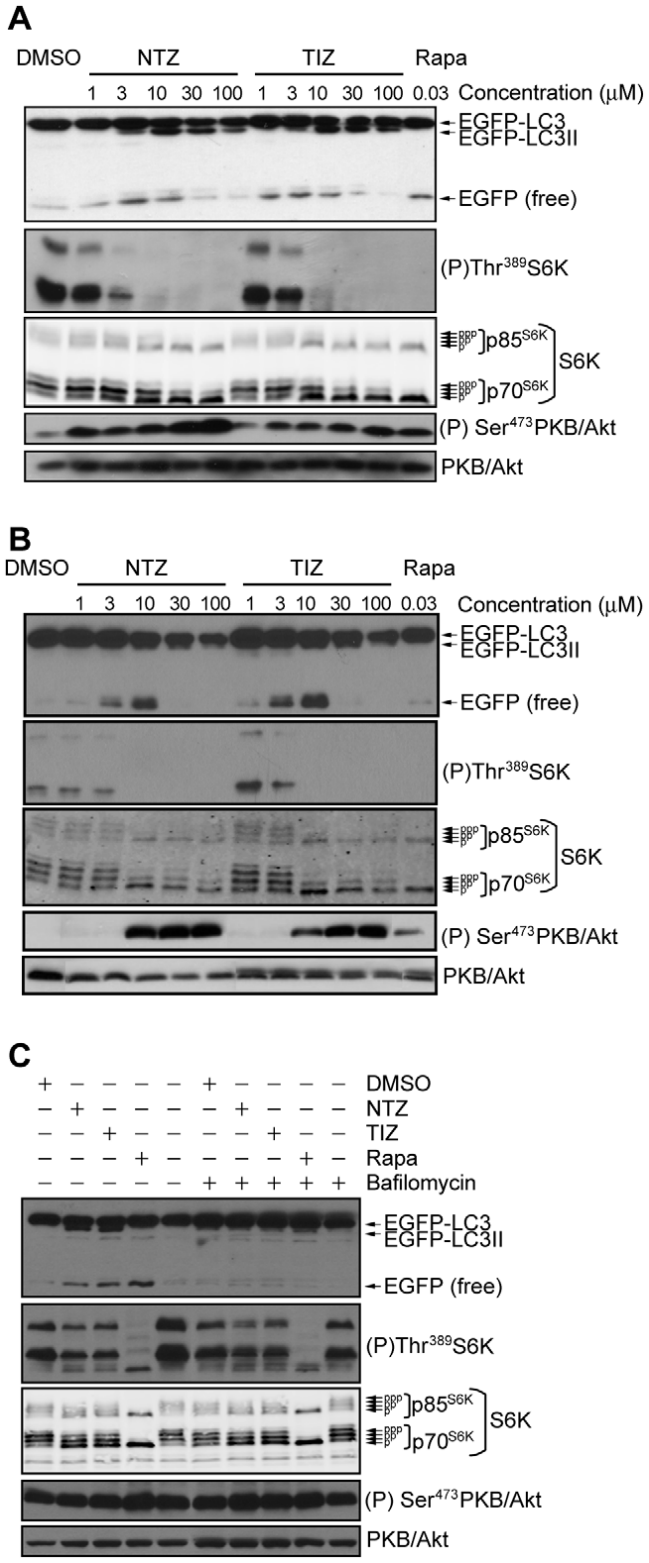
Increased EGFP-LC3 processing and inhibition of mTORC1 signaling by NTZ and TIZ. Cells were treated with the indicated concentrations of NTZ, TIZ or rapamycin for 4 h (A) or 24 h (B). In panel C, cells were treated with 10 µM NTZ, 10 µM TIZ, 30 nM rapamycin, or DMSO without or with 0.1 µM bafilomycin A1 for 4 h. EGFP-LC3 processing was examined by immunoblotting with antibodies against GFP, mTORC1 activity using antisera against phospho-S6K Thr^389^ and total S6K, and mTORC2 activity with antisera against phospho-AKT Ser^473^ and total AKT. Total AKT was also used as a loading control.

Untreated cells showed mostly full length EGFP-LC3 and only a small amount of free EGFP (Figure 2A). Incubation with NTZ or TIZ for 4 h (Figure 2A) and 24 h (Figure 2B) caused a concentration-dependent increase in the lipidated product EGFP-LC3II that was visible at 3 µM and strong at ≥10 µM. These results show the drugs increased the processing of EGFP-LC3 that accompanies autophagosome formation. NTZ and TIZ also caused an increase in the free EGFP band (Figure 2A, B) that was abolished in the presence of the vacuolar-type H^+^ ATPase inhibitor bafilomycin A1 (Figure 2C), a known inhibitor of lysosomal fusion [33], [34]. This shows that NTZ and TIZ can increase autophagic flux. Concentrations of NTZ and TIZ ≥30 µM resulted in decreased free EGFP, despite the presence of an EGFP-LC3II band (Figure 2A,B), raising the possibility that autophagic flux was reduced at high concentrations of NTZ and TIZ.As discussed further below, a decrease could be a consequence of activation of PKB/Akt (Figure 2A, B), which is known to downregulate autophagy [35], [36].

### Nitazoxanide inhibits mTORC1 but not mTORC2 signaling

mTORC1 is a protein kinase complex whose activity may be monitored by measuring phosphorylation of key substrates such as ribosomal S6 kinases (S6Ks) by immunoblotting [37]. Cells maintained in complete cell culture medium containing serum and nutrients showed high levels of Thr^389^ phosphorylation by mTORC1 and increased electrophoretic mobility of p70^S6K^andp85^S6K^(Figure 2A) and this was completely repressed within 4 h exposure to the mTORC1 inhibitor rapamycin (Figure 2A). Exposure to different concentrations of NTZ or TIZ for 4 h or 24 h resulted in a concentration-dependent decrease inS6K phosphorylation - inhibition was partial at 1 µM, strong at 3 µM and essentially complete at 10 µM(Figure 2A,B). Thus, NTZ and TIZ inhibit mTORC1 signaling.

mTOR is also the catalytic subunit of a second complex termed mTORC2 that phosphorylates PKB/Akt at Ser^473^ [38]. The rapamycin insensitive mTORC2 modulates changes in the cytoskeleton and is largely unaffected by nutrients or energy conditions [39]. To determine whether NTZ and TIZ inhibit mTORC2, the same cell lysates were probed with a PKB/Akt Ser^473^ phosphospecific antibody. Untreated cells displayed mTORC2 activity that was not inhibited by rapamycin(Figure 2A, B). In fact, rapamycin caused an increase in mTORC2 activity (Figure 2A, B). This is consistent with previous studies showing that mTORC1/S6K1 signaling downregulates PKB/Akt phosphorylation on Ser^473^ via a feedback loop involving transcriptional inhibition of the insulin receptor substrate IRS-1 gene and degradation of IRS-1 and IRS-2 proteins, and that mTORC1 inhibition prevents the establishment of this feedback loop, thus increasing PKB/Akt Ser^473^ phosphorylation [40], [41]. NTZ and TIZ also did not decrease PKB/Akt Ser^473^ phosphorylation but rather increased it in a concentration-dependent manner that paralleled their level of mTORC1 inhibition (Figure 2A, B).The increase in PKB/AKT Ser^473^ phosphorylation at higher concentrations of NTZ and TIZ was concomitant with the decrease in free EGFP (Figure 2A, B). Since PKB/Akt downregulates autophagy [42], it is possible that at high concentrations of NTZ and TIZ and long incubation times, inhibition of mTORC1 stimulates autophagy while feedback activation of PKB/Akt elicits signals that counteract autophagy.

**Figure 3:**
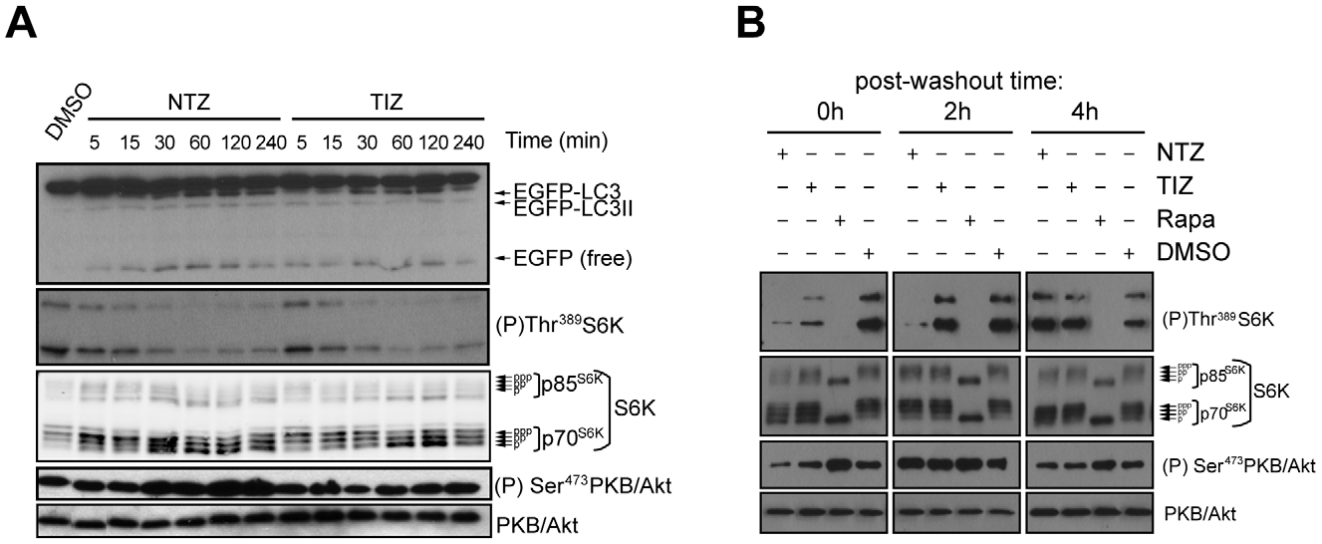
Time-course and reversibility of mTORC1 inhibition and EGFP-LC3 processing by NTZ and TIZ. (A) Cells were incubated with 10 µM NTZ or 10 µM TIZ for the indicated times. (B) Cells were incubated with 10 µM NTZ, 10 µM TIZ or 30 nM rapamycin for 4 h. The drugs were then washed away and cells were incubated in drug-free medium for the indicated times post-washout. Cell lystates were immunoblotted as in Figure 2.

Inhibition of mTORC1 signaling was detectable after 30 min exposure to 10 µM NTZ or TIZ and maximal after ≥1 h (Figure 3A) and PKB/Akt Ser^473^ phosphorylation rose detectably at 30 min and increased further at longer exposure times (Figure 3A). To examine whether mTORC1 signaling inhibition was reversible, cells were incubated with 10 µM NTZ or TIZ for 4 h, the drugs were washed away and the cells were incubated in drug-free medium for different times. As a comparison, cells were treated with rapamycin, which inhibits mTORC1 essentially irreversibly. mTORC1 was strongly inhibited after 4 h exposure to either NTZ or TIZ (Figure 3B, 0 h) but its activity increased over time after drug removal, returning to levels seen in untreated cells within 4 h, while mTORC1 activity remained profoundly inhibited upon rapamycin withdrawal (Figure 3B).

Together, these results show that NTZ and TIZ strongly but reversibly inhibit mTORC1 signaling. Since the mTOR catalytic subunit is shared by mTORC1 and mTORC2, the observation that the drugs inhibit mTORC1 while activating mTORC2 is strong evidence that they do not directly inhibit the kinase activity of mTOR itself but likely act on an upstream mTORC1 regulatory pathway.

### Structural requirements for stimulation of autophagy and inhibition of mTORC1 by nitazoxanide

**Figure 4:**
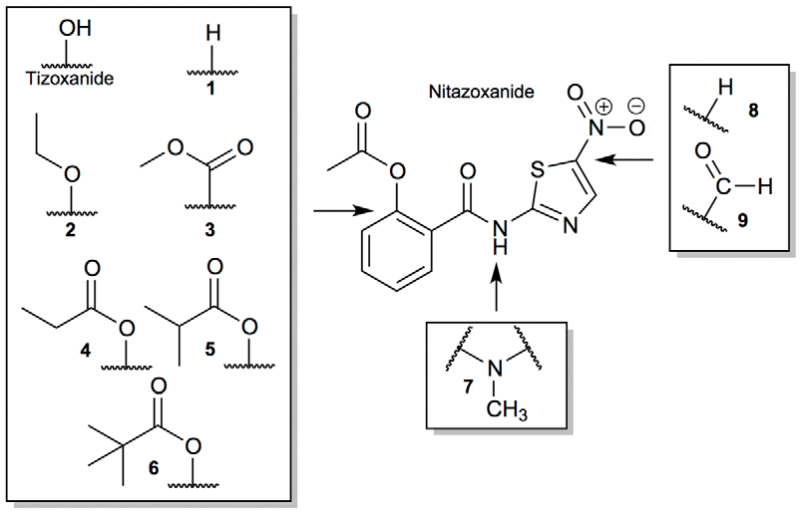
Structures of NTZ analogues.

NTZ targets in mammals are not well-characterized. To address its mechanism of action in human cells, we first carried out a limited structure-activity relationship study. Sixteen analogues (Figure 4 and Figure S2) were synthesized and tested for effects on autophagosome accumulation, EGFP-LC3 processing and inhibition of mTORC1 activity (Table 1).

**Table 1:** Effect of Nitazoxanide analogs (10 µM) on autophagosome induction, EGFP-LC3 and mTORC1 inhibition.

Analogue	Autophagosome formation	EGFP-LC3 processing	mTORC1 inhibition
DMSO	−	−	−
NTZ	+++	+++	+++
TIZ	+++	+++	+++
**1**	−	−	−
**2**	−	−	−
**3**	−	−	−
**4**	++	+	+
**5**	+++	+++	+++
**6**	++	+	+
**7–16**	−	−	−

NTZ and TIZ(Figure 4) showed similarly strong activity in all three assays (Table 1). Analogue **1** lacking the OH group was completely inactive in all three assays, implying that generation of the OH group by hydrolysis of NTZ is essential for activity. In support of this point, analogue **2** with an ether bond that is not cleavable by esterases was inactive in all three assays (Table 1). In addition, analogue **3** bearing a methylacetate substituent like NTZ but arranged such that cleavage by esterases would generate a carboxylic acid instead of an OH, was also inactive in all assays. Moreover, analogues **4**, **5** and **6**, bearing more bulky propionate, isobutyrate and pivalate substituents that are cleaved more slowly by esterases, were also active in all three assays. Together, these data show that NTZ is the active prodrug of TIZ and that the OH group is critical for activity.

Methylation of the NH group in the linker region (compound **7**, Figure 4) caused complete loss of activity, suggesting that inter- or intramolecular hydrogen bonding by the secondary amine or the generation of an anionic form of NTZ [43] is required for activity. Nitazoxanide also possesses a conspicuous nitro group attached to the 5-membered ring (Figure 4). Analogue **8** lacking this group was completely inactive in all three assays, showing the nitro group is also required for activity. Analogue **9** with an aldehyde substituent was also inactive, indicating that the strongly electron-withdrawing character of the nitro group is more relevant to activity than its steric bulk.

Addition of one or two chlorine substituents to the 6-membered ring of NTZ also eliminated activity. Since niclosamide possesses a Cl substituent para to the OH group, this result indicates that NTZ and niclosamide probably have different cellular targets. Several combinations of substitutions described individually above were also inactive in all assays(Table 1, Figure S1).

Together, these results show that the OH, NH and NO_2_ groups are all essential for induction of autophagosome accumulation, EGFP-LC3 processing and mTORC1 inhibition, revealing strict structural requirements for activity. The tight correlation between the structural features required for activity in all three assays also strongly implies that these biological responses are linked and probably result from inhibition of a single target rather than multiple independent targets.

### Nitazoxanide and tizoxanide inhibit intracellular Mtb proliferation

**Figure 5:**
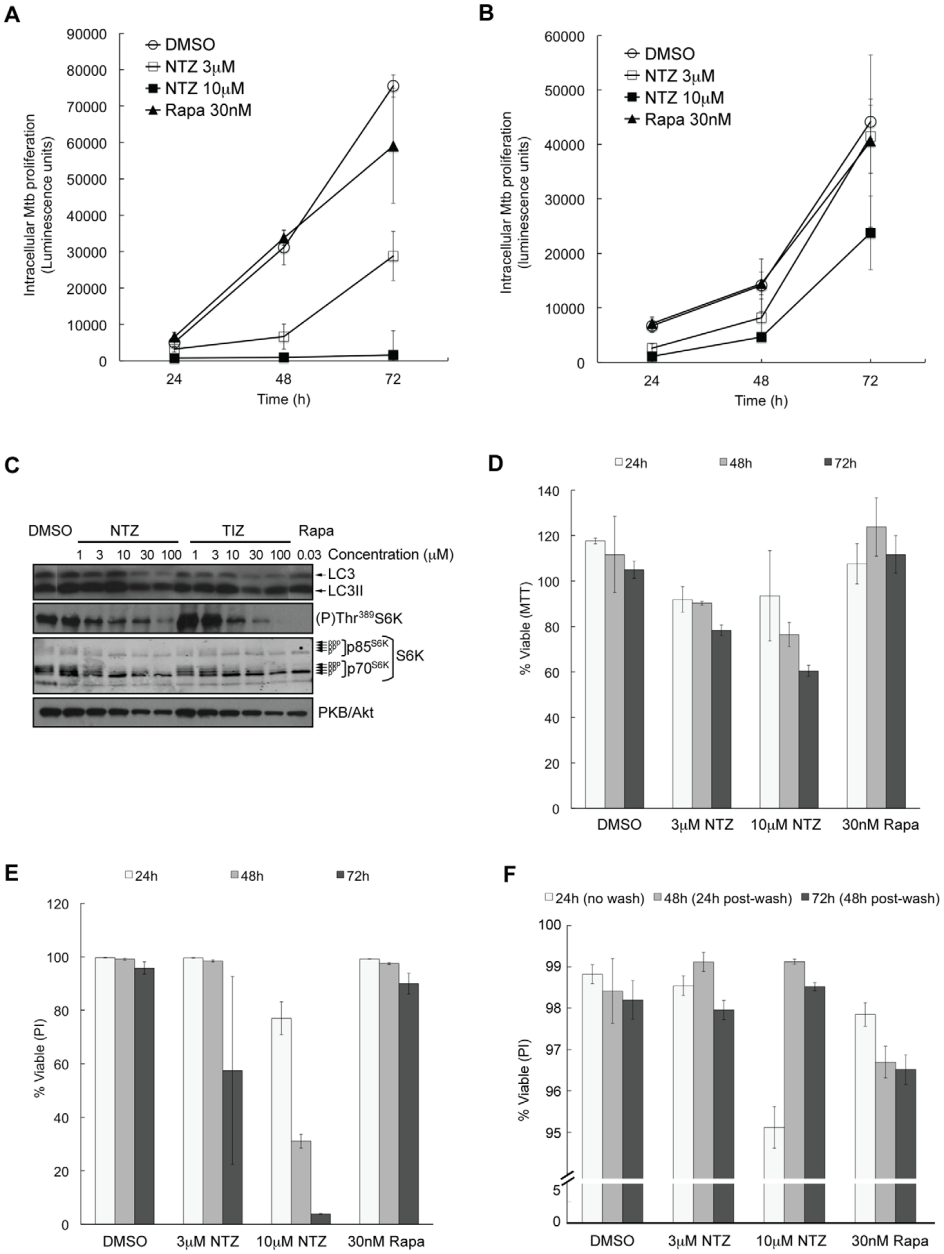
Effect of NTZ on Mtb proliferation and survival of THP-1 cells. (A) Differentiated THP-1 cells infected with Mtb H37Rv bearing a luciferase-reporting plasmid were treated with various concentrations of NTZ or TIZ for the indicated times. Intracellular Mtb was measured as luciferase activity at 24, 48 and 72 h. (B) Infected THP-1 cells were treated as in (A) but after 24 h treatment the drugs were removed and cells were incubated with medium without drug. Luciferase activity was measured at 24 h, 48 h (24 h post-wash) and 72 h (48 h post-wash). (C) Differentiated THP-1 cells were exposed to various concentrations of NTZ, TIZ or rapamycin for 4 h. Endogenous LC3 processing (using LC3 antibodies) and mTORC1 activity were determined by immunoblotting as in Figure 2.Viability of THP-1 cells treated with drugs for the indicated times was measured with the MTT assay (D) or by propidium iodide (PI) staining (E, F). PI levels were measured quantitatively by automated microscopy, and viable cells was calculated as the percentage of PI-negative cells. (F) After drug removal at 24 h, THP-1 cell survival was measured by PI at 48 h (24 h post-wash) and 72 h (48 h post-wash).

We next used a luciferase reporter assay to assess the ability of NTZ to inhibit Mtb growth in infected macrophages. Differentiated THP-1 human acute monocytic leukemia cells were infected with a Mtb strain expressing luciferase. After removal of non-internalized bacteria, NTZ was added and Mtb proliferation was determined 24, 48, and 72 h later. NTZ showed concentration-dependent inhibition at all time points, with essentially complete inhibition at 10 µM (Figure 5A). Exposure of Mtb in liquid culture to 10 µM NTZ was able to reduce Mtb growth, albeit less potently compared to the growth in THP-1 cells (Figure S3).

To examine the reversibility of the effects of NTZ, infected THP-1 cells were treated with NTZ for 24 h after which time the drug was removed and cells were cultured for up to two additional days. Exposure to 3 µM or 10 µM NTZ significantly reduced Mtb proliferation at 24 h but intracellular Mtb proliferation resumed upon drug removal (Figure 5B). Immunoblotting for endogenous LC3 and for phospho-Thr^389^S6K indicated that autophagy was induced by NTZ and TIZ in THP-1 cells, and that mTORC1 was inhibited (Figure 5C). Rapamycin, which does not inhibit all functions of mTORC1 and is a weak stimulator of autophagy [44], [45], had no effect on Mtb proliferation in THP-1 cells (Figure 5A, B, and Figure S4) or in liquid culture in the absence of cells (Figure S3),had only a modest effect on the conversion of LC3 to LC3II, while completely inhibiting Thr^389^S6K phosphorylation (Figure 5C), and did not affect THP-1 cell survival (Figure 5D–F).

We also assessed the effect of NTZ on the viability of differentiated THP-1 cells using three different assays: the MTT assay to measure cell metabolic activity (Figure 5D), propidium iodide (PI) exclusion to measure plasma membrane integrity (Figure 5E),and staining of live cells with Hoechst 33342 to count attached cells. The MTT assay showed a modest time- and concentration-dependent reduction in THP-1 cell metabolic activity (Figure 5D), as did the Hoechst 33342 assay (unpublished data). By contrast, after 72 h of treatment with 10 µM NTZ, large numbers of cells had taken up PI (Figure 5E).Cells treated with NTZ for 24 h and transferred to drug-free medium retained viability (Figure 5F).

The observation that after 72 h 10 µM NTZ had only minor effects in the MTT assay but strongly increased PI uptake in differentiated THP-1 cells was perplexing. Cellular toxicity was unexpected given the knowledge that peak plasma concentrations of approximately 37 µM are achieved after a standard oral dose of 500 mg NTZ [30] and that NTZ has an excellent safety profile, with no significant side effects in patients taking 500 mg NTZ twice daily for 24 weeks [46]. We therefore considered it important to examine the effects of NTZ on human primary peripheral blood mononuclear cells (PBMC), as cells of more direct relevance to Mtb therapy.

**Figure 6:**
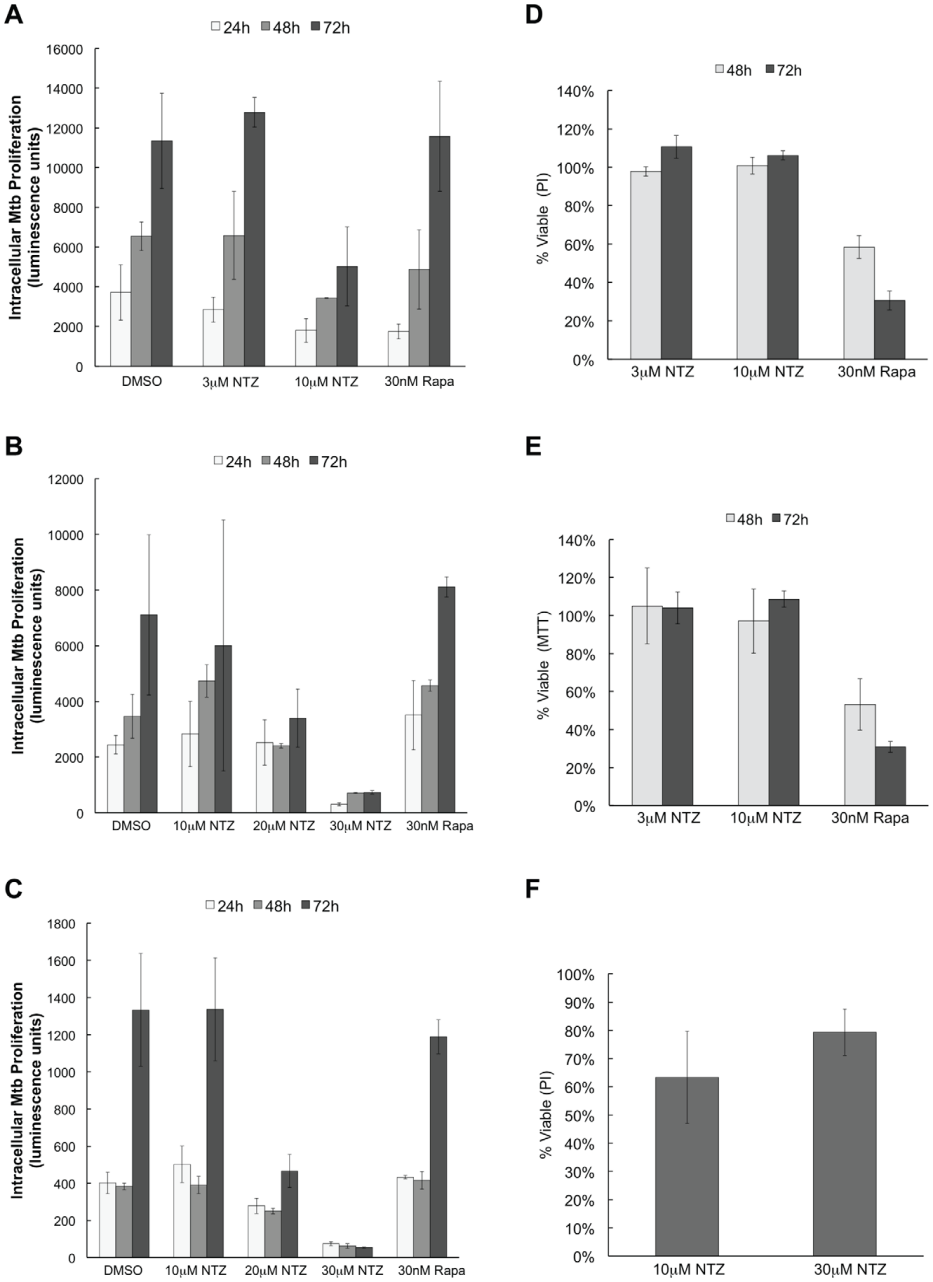
Effects of NTZ on Mtb proliferation and viability of PBMC. Peripheral blood mononuclear cells isolated from healthy human subjects and infected with Mtb H37Rv bearing a luciferase-reporting plasmid were treated with various concentrations of NTZ for the indicated times. Intracellular Mtb was measured as luciferase activity. Data presented are: subject 1withMOI 10 (A), subject 2with MOI 1 (B) and subject 2 with MOI 10 (C). Viability normalized to DMSO-treated controls was measured at 48 and 72 h, with PBMC from subject 2 using the MTT assay (D) or by PI staining (E).(F) Viability, normalized to DMSO treated controls, was measured with PBMC from subject 3 at 72 h.

PBMC were isolated from blood samples from a number of healthy human donors. Cells were infected with Mtb at a multiplicity of infection (MOI) of 1 or 10 for 24 h, after which non-internalized bacteria were washed away and infected cells were exposed to 3–30 µM NTZ for up to 72 h. The effect of 10 µM NTZ on intracellular Mtb proliferation ranged from 0 to 55% inhibition at 72 h, depending on the donor and the multiplicity of infection (Figure 6A–C). 30 µM NTZ almost completely inhibited Mtb proliferation, starting as early as 24 h (Figure 6B, C).Notably, exposure of PBMC to NTZ at 10 µM or 30 µM for up to 72 h caused no cytotoxicity, with cell survival remaining at 100%, in both the PI uptake and MTT assays (Figure 6D–F). By contrast, rapamycin reduced cell survival to ∼30% (Figure 6D–E).

These observations show that NTZ can inhibit the intracellular proliferation of Mtb at concentrations that are not toxic to primary human cells. They are consistent with previous observations that rapamycin has toxic effects on primary human cells [47]and that NTZ is safe in humans at a plasma concentration of 30 µM [30], [48].

### Nitazoxanide inhibits the human NAD(P)H quinone oxidoreductase NQO1

**Figure 7:**
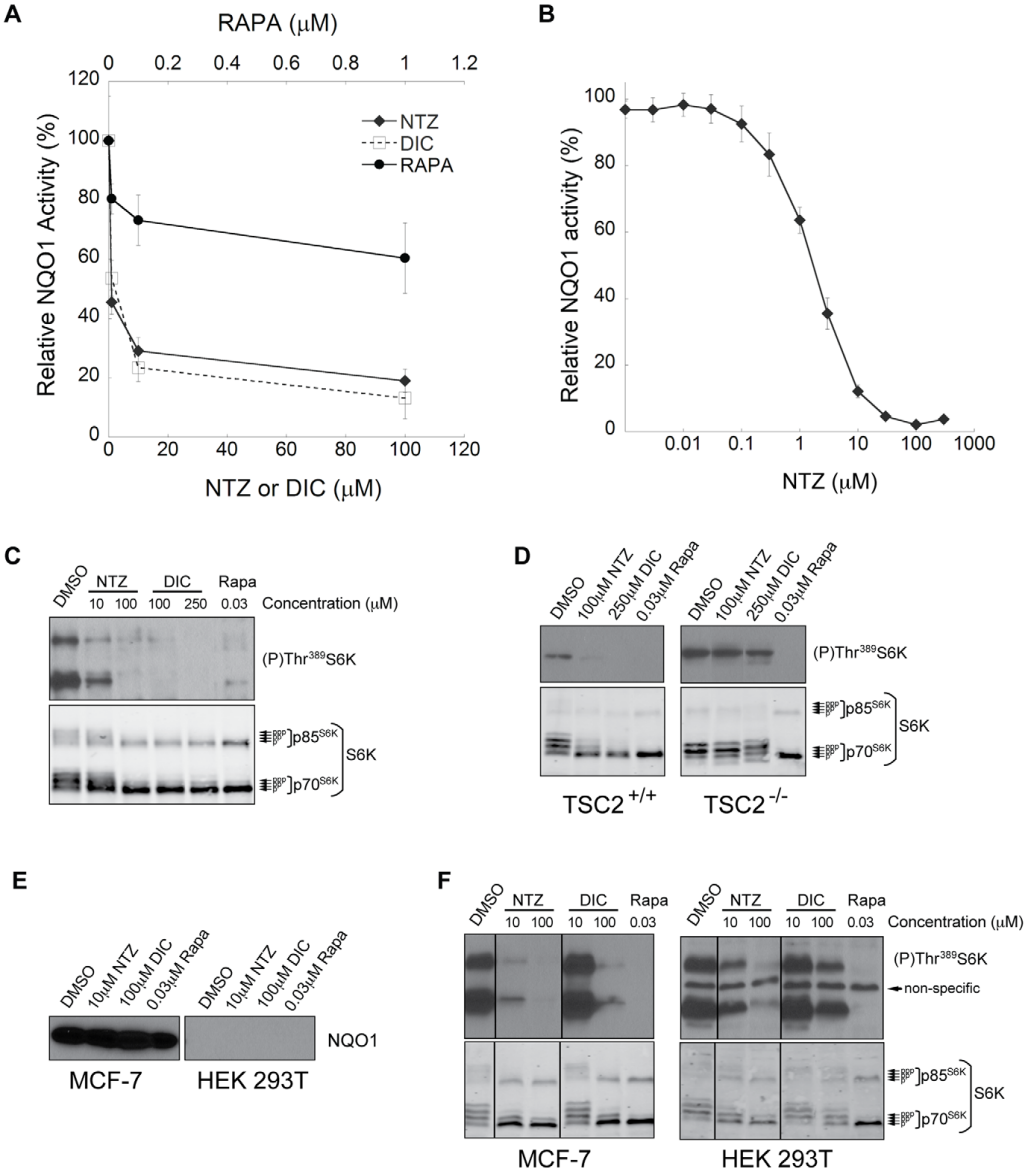
Inhibition of NQO1 by NTZ and TSC2-dependent mTORC1 inhibition. (A) Relative activity of NQO1 as determined by a modified Prochaska microtiter plate bioassay, in which 0.25 µg/ml pure NQO1 enzyme was treated with different concentrations of NTZ, dicoumarol (DIC), and rapamycin (RAPA) for 1 h. (B) MCF-7 cell lysates were treated with various concentrations of NTZ for 1 h, and relative activity of NQO1 was determined by the Prochaska bioassay. (C) MCF-7 cells or (D) TSC2^+/+^ and TSC2^−/−^MEFs were treated with NTZ, DIC, and rapamycin for 4 h and subjected to immunoblotting for total and phosophorylated S6K as previously described.(E) Total NQO1 or (F) total and phosphorylated S6K was assessed by immunoblotting in MCF-7 or HEK 293T cells treated with indicated concentrations of drugs for 8 h. Lines in (F) were used to indicate juxtaposition of noncontinguous lanes from the same gel and image exposure.

Nitazoxanide has no known targets in humans. Our results thus far suggest that it may target an upstream mTORC1 regulatory pathway rather than acting directly on mTORC1 or autophagy. It has recently been observed using affinity chromatography that RM4847, a bromo derivative of TIZ, can bind human quinone oxidoreductase NQO1 [49]. We used a modified version of the Prochaska microtiter assay to monitor the effects of NTZ on NQO1 activity [50], [51]. NTZ was able to inhibit purified NQO1 *in vitro* in a concentration-dependent manner, as did dicoumarol (DIC), a known competitive inhibitor of NQO1 enzymatic activity [52] (Figure 7A). Rapamycin, at a concentration that completely inhibits mTORC1(0.1 µM),did not cause significant NQO1 inhibition, and NQO1 remained 60% active at a very high concentration of rapamycin (1 µM) (Figure 7A). Next, we performed the Prochaska assay on freshly prepared MCF-7 cell lysates. Cellular NQO1 activity was reduced to approximately 14% by 10 µM NTZ (Figure 7B), a concentration that inhibits mTORC1 in cells.

We also observed that the NQO1 inhibitor DIC can strongly inhibit mTORC1, as assessed by S6K phosphorylation at 4 h (Figure 7C), raising the possibility that NQO1 may be an upstream regulator of mTORC1. The TSC1–TSC2 tuberous sclerosis complex is a major negative upstream regulator of mTORC1 and mouse embryo fibroblasts (MEFs) lacking the TSC2 gene (TSC2^−/−^) show high mTORC1 activity compared to TSC2^+/+^MEFs [53]. NTZ and DIC inhibited mTORC1 in TSC2^+/+^ cells but not in TSC2^−/−^ cells (Figure 7D), indicating that inhibition is dependent on the TSC1–TSC2 complex. We also examined the effects of NTZ on HEK 293T cells, which do not express NQO1 at a detectable level (Figure 7E). Concentrations of NTZ or DIC that significantly inhibited mTORC1 in MCF-7 cells, which express a much higher level of NQO1 protein (Figure 7E),did not exert as great an effect on mTORC1 signaling in HEK 293T cells (Figure 7F). These data are consistent with inhibition of NQO1 leading to downstream mTORC1 inactivation by a pathway involving the TSC1–TSC2 complex. By contrast, rapamycin inhibited mTORC1 equally in both cell lines (Figure 7D–F). The different cellular targets of rapamycin and NTZ may explain why rapamycin was unable to inhibit intracellular Mtb proliferation in infected cells.

### Tuberculosis drugs do not interfere with the ability of NTZ and TIZ to induce autophagosome formation

**Figure 8:**
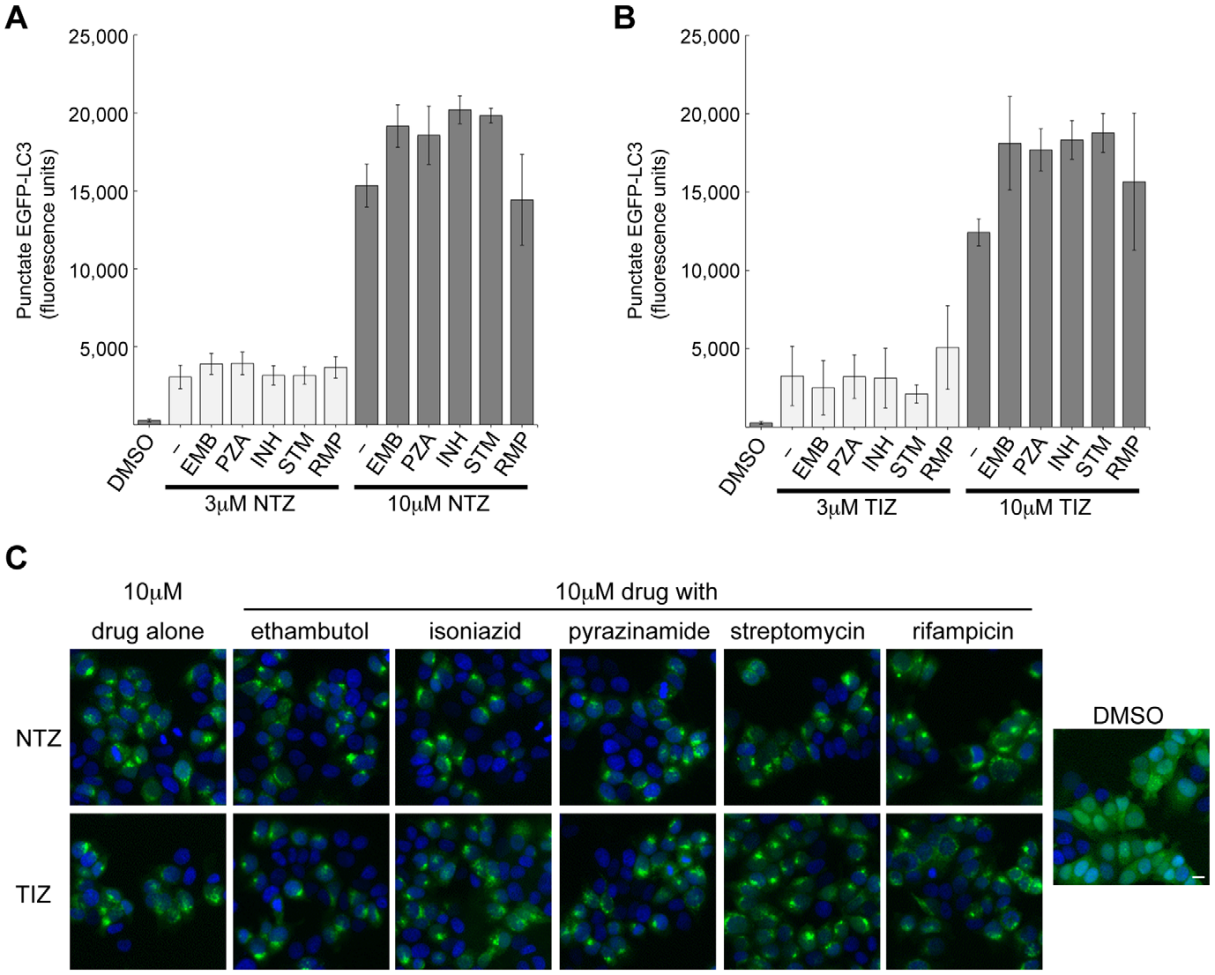
Effect of NTZ and TIZ on autophagosome formation in the presence of tuberculosis drugs. MCF-7 cells expressing EGFP-LC3 were exposed to 3 µM or 10 µM NTZ (A) or TIZ (B), without or with 5 µg/ml ethambutol (EMB), pyrazinamide (PZA), isoniazid (INH), streptomycin (STM) or rifampicin(RMP). Representative images of treated cells are shown in (C). Scale bar, 10 µm.

Tuberculosis is always treated with drug combinations. We therefore asked whether NTZ and TIZ retain their ability to induce autophagosomes in the presence of tuberculosis drugs. MCF-7 cells were exposed to ethambutol (EMB), isoniazid (INH), pyrazinamide (PZA), streptomycin (STM) or rifampicin (RMP)aloneor with NTZ or TIZ. None of the drugs, when tested between 0.5 µg/ml and 50 µg/ml(equivalent to 2.45 µM and 244.73 µM EMB; 1.37 µM and 137.14 µM INH; 4.06 µM and 406.13 µM PZA; 0.86 µM and 85.97 µM STM; and 0.61 µM and 60.76 µM RMP), prevented the induction of punctate EGFP-LC3 by NTZ or TIZ at 3 or 10 µM (Figure 8). These results predict that NTZ would retain its ability to affect autophagy when used in combination treatment with tuberculosis drugs.

## Discussion

This study reveals new cellular roles for the antiparasitic drug nitazoxanide and its active metabolite tizoxanide as stimulators of autophagy and inhibitors of mTORC1 signaling, acting at least in part through suppression of the quinone oxidoreductase NQO1. Our work also demonstrates that these drugs inhibit Mtb proliferation in human cells. NTZ is a noncompetitive inhibitor of pyruvate: ferredoxin oxidoreductase (PFOR) [43], an enzyme found in amitochondriate human parasites and most anaerobic bacteria but that is not conserved in mammals. PFOR catalyses the oxidative decarboxylation of pyruvate to acetyl coenzyme A and CO_2_ [43], [54] whereas mammals oxidize pyruvate using pyruvate dehydrogenase, which is not inhibited by NTZ [55]. To our knowledge, NTZ has not previously been shown to have direct targets in human cells or to act on host cell signaling pathways.

The thiazolide RM4847 differs from TIZ in having a Br substituent instead of a nitro group as well as methyl groups attached to the 6-membered ring and to the 5-membered ring. RM4847 coupled to agarose beads has been observed to bind human glutathione-S-transferase, GSTP1 [56] and human NQO1 [49]. We did not observe inhibition of cellular GSTP1 by NTZ (unpublished data) using the 1-chloro-2,4-dinitrobenzene reduction GSTP1 enzymatic activity assay [57], [58]. However, NTZ inhibited cellular NQO1 enzymatic activity in a concentration-dependent manner, strongly reducing NQO1 activity at 10 µM. Moreover, DIC, an NQO1 inhibitor, also inhibited mTORC1 signaling. The reduction of NQO1 enzymatic activity likely contributes to attenuation of mTORC1 signaling, since deletion of the major mTORC1 regulator TSC1–TSC2 rendered cells unresponsive to the effects of NTZ or DIC.NTZ and DIC were also less potent inhibitors of mTORC1 in cells that do not express NQO1, indicating that NQO1 is a relevant cellular target, but probably not the only one. Thus, NTZ may stimulate autophagy and inhibit mTORC1 signaling and intracellular Mtb proliferation at least in part as a result of inhibiting NQO1.

NQO1 can catalyze the reduction of a broad range of reactive substrates including quinones, quinone-imines and nitro-compounds to less reactive and less toxic forms [59]. It has also been shown to be a superoxide scavenger [60] and a “gatekeeper” of the 20S proteasome [61], [62]. We speculate that NQO1 may be linked to autophagy throughp62/sequestosome-1, an adaptor protein that binds LC3 and ubiquitin to facilitate autophagy of polyubiquitinated proteins [63]. This speculation is based on the observations that p62 canactivate the redox-sensing transcription factorNrf2resulting in persistent expression of NQO1 [64] and that knockdown of Nrf2caused a 2-fold increase in autophagy [65]. Our data linking NQO1 activity and mTORC1 signaling are in line with studies showing that NQO1 expression is induced to counter the toxicity of elevated levels of reactive oxygen species (ROS) [66], and that increased oxidative stress from elevated ROS inhibits mTORC1 signaling and induces autophagy through activation of TSC2 [67].Pharmacological inhibition of NQO1 may increase oxidative stress to inhibit mTORC1 and activate autophagy, thereby inhibiting intracellular Mtb proliferation.

NTZ can directly kill both replicating and nonreplicating Mtb *in vitro* [68]. NTZ kills Mtb *in vitro* in liquid and solid media at a minimum inhibitory concentration of ∼50 µM after 7 days [68]. This occurs by an uncharacterized mechanism because PFOR is not known to be present in Mtb [68]. This observation raises the question of whether NTZ prevents the replication of Mtb in human cells solely by targeting the bacteria themselves or also by affecting a host cellular process such as autophagy. In our hands, 10 µM NTZ completely inhibited mTORC1 signaling and strongly stimulated autophagy while this concentration directly inhibited Mtb proliferation in liquid culture less robustly (Figure S3). Therefore it can be argued that concentrations of NTZ that directly kill Mtb would also necessarily affect autophagy, a host cellular process implicated in Mtb intracellular proliferation. Additionally, 10 µM NTZ inhibited the intracellular replication of Mtb in THP-1 cells more strongly than it inhibited Mtb directly. The interpretation of this latter result is complicated by the observation that the PI exclusion assay, but not the MTT or Hoechst assays, showed 10 µM NTZ to be significantly toxic to differentiated THP-1 cells at longer exposure times. More importantly, all assays showed NTZ was not toxic to PBMC at the highest concentration and exposure time tested (30 µM, 72 h), consistent with its very safe toxicity profile in humans. Indeed, a peak plasma concentration of 37 µM TIZ was observed after a standard single oral dose of 500 mg NTZ while1 g administered twice daily for one week resulted in peak plasma concentrations reaching 100 µM, and neither was associated with adverse side effects [30]. Therefore, the toxicity of NTZ towards the differentiated THP-1 cell line in culture does not appear to reflect the *in vivo* situation. NTZ also inhibited the intracellular proliferation of Mtb in PBMC. Significant inhibition required a concentration of 10–30 µM (Figure 6), the range reflecting different sensitivities of PBMC from different donors.

In our experiments, NTZ and rapamycin both efficiently inhibited mTORC1 but NTZ reduced intracellular Mtb proliferation while rapamycin did not. This result is in apparent contrast with a previous report that rapamycin reduced Mtb replication in RAW 264.7 cells [19]. Different experimental conditions may explain this discrepancy, including the use of a much higher rapamycin concentration of 50 µM. Here, we used 30 nM, which was sufficient to completely inhibit the phosphorylation of S6K by mTORC1 in THP-1 cells. A more recent study reported ∼50% reduction in Mtb colony-forming units after 4 h treatment of infected THP-1 cells with 27 µM rapamycin [69].

Anti-tuberculosis drugs are limited in number and efficacy and prone to many side effects and are often inactive against nonreplicating (dormant) Mtb. Furthermore, multi drug-resistant strains are resistant to the first-line drugs rifampicin and isoniazid and extensively drug-resistant strains are also resistant to any fluoroquinolone and any of the second-line injectable drugs, and give very high mortality rates [2], [70]. Even non-resistant strains of Mtb can require up to nine months of therapy with a combination of at least three antibiotics to reduce development of resistance [71], [72]. Therefore new drugs with improved efficacy, shorter treatment time and lower cost are urgently needed.

NTZ is a safe, effective, orally bioavailable and inexpensive drug already approved for human use. It has shown broad-spectrum activity against anaerobic intestinal helminths and protozoans as well as some bacteria, including *Clostridium difficile* [73]–[79] and is approved for the treatment of enteritis caused by *Cryptosporidium parvum* and *Giardia intestinalis* [29]. The length of NTZ treatment is typically 3–14 days, but it has been used for up to 4 years in AIDS-related cryptosporidiosis patients without significant adverse effects [77], [80] and resistance to NTZ has not been reported. NTZ inhibited mTORC1, modulated autophagy, inhibited NQO1 and inhibited Mtb proliferation in PBMC at concentrations well within tolerated plasma concentrations. The autophagy-stimulating effect of NTZ was not impaired in the presence of the anti-tuberculosis drugs ethambutol, isoniazid, pyrazinamide, streptomycin, or rifampicin. Drugs such as NTZ that both directly inhibit the proliferation of pathogenic microorganism and stimulate host cellular defense mechanisms such as autophagy may provide new opportunities to combat intracellular pathogens like Mtb.

## Materials and Methods

### Ethics statement

Peripheral blood was collected from normal human subjects, in accordance with the ethics approval guidelines of the University of British Columbia Research Ethics Board and Tri-Council Policy Statement for Ethical Conduct for Research Involving Humans, for secondary use of anonymous human blood. Written informed consent was obtained from all study participants.

### Reagents

Cell culture reagents were purchased from Invitrogen, unless stated otherwise. General laboratory chemicals were purchased from Sigma-Aldrich, Fisher Scientific and BDH Inc. Nitazoxanide (N490100) and tizoxanide (T450100) were purchased from Toronto Research Chemicals Inc, rapamycin (553210) from Calbiochem, and Hoechst 33342 (H3570) from Invitrogen. Dicoumarol (Sigma M1390) was prepared fresh in DMSO before each experiment. Anti-phospho Thr389 S6K (#9205), anti- phospho Ser473 Akt (#9271), anti-Akt (#9272), and anti-NQO1 (#3187) antibodies were from Cell Signaling Technology. Anti-S6K C-18 (#230) antibody was purchased from Santa Cruz Biotechnology, and anti-GFP antibody (#1814460) was from Roche. Antibody to endogenous LC3 was purchased from Nanotools (0260-100). The *M. tuberculosis* H37Rv strain carrying pMV361-c1-lucplasmid was used for macrophage infection studies. pMV361-c1-luc carries a 1.65 kb fragment of the firefly luciferase gene under constitutive expression of a mycobacterial HSP60 promoter [81].

### Cell culture procedures

MCF-7 cells stably transfected with pEGFP-LC3 have been described previously [28]. These cells were maintained in RPMI-1640 medium (Invitrogen 72400) supplemented with 400 µg/mL G418 (Promega #V7982) and 10% (v/v) fetal bovine serum (FBS) with 2 g/L sodium bicarbonate and 1 mM HEPES. THP-1 cells were maintained in RPMI-1640 supplemented with 10% FBS and 1% glutamine.TSC2^−/−^/p53^−/−^ and TSC2^+/+^/p53^−/−^ mouse embryo fibroblasts (MEF) were a generous gift of Dr. David Kwiatkowski [82]. MEF and HEK 293T cells were maintained in high glucose Dulbecco's modified Eagle's medium (DMEM) supplemented with 10% FBS.

### Automated assay for autophagosome induction

MCF-7 cells stably expressing EGFP-LC3 were seeded at 20,000 cells/well in PerkinElmer View 96-well plates. Eighteen hours after seeding, chemicals were added to each well and plates were incubated at 37°C for the indicated times. The medium was removed and cells were fixed with 3% (v/v) paraformaldehyde containing 500 ng/mL Hoechst 33342 for 15 min at room temperature. Fixed cells were washed once with PBS containing 1 mM MgCl_2_ and 0.1 mM CaCl_2_ and stored in the same medium at 4°C overnight. Plates were scanned using a Cellomics™Arrayscan VTI automated fluorescence imager. Cells were photographed and quantified using the compartment analysis algorithm as previously described [28].

### Cell lysis and protein quantification

Cells were harvested in 20 mM Tris-HCl pH 7.5, 150 mM NaCl, 1 mM EDTA, 1 mM EGTA, 1% (v/v) Triton X-100, 2.5 mM sodium pyrophosphate, 1 mM β-glycerophosphate supplemented with fresh 1 mM Na_3_VO_4_, 1 mM dithiothreitol, and 1× complete protease inhibitor cocktail (Roche, #1169748001). Supernatants were collected after centrifugation at 18,000×g for 15 min at 4°C and quantified using the BCA protein assay kit (Pierce #23227).

### SDS-PAGE and immunoblotting

Electrophoresis and immunoblotting conditions for EGFP-LC3 processing, S6K and AKT in MCF-7, THP-1 and HEK 293T cells were exactly as previously described [28]. MEF were seeded in 6-well plates at 400,000 cells/well (TSC2^+/+^) or 200,000 cells/well (TSC2^+/+^) in normal DMEM medium, cultured overnight and treated similarly.NQO1 levels were assessed by resolving protein samples on a 10% acrylamide gel and subjected to electroblotting onto nitrocellulose, then blocked with 5% (w/v) fat-free milk and incubated with NQO1 antisera. For immunoblotting of endogenous LC3, proteins were transferred onto polyvinylidene fluoride membrane with membrane with 0.2 µm pores, which were then fixed with 0.2% glutaraldehyde in PBS with 0.02% Tween-20 for 20 min at room temperature, prior to blocking with 5% fat-free milk.

### Synthesis of nitazoxanide analogues

Analogues **1**, **10**, **11** were prepared by coupling between benzoyl chloride and heteroaromatic primary amines. Analogues **2**, **12**, **13** were prepared by coupling between 2-ethoxybenzoyl chloride and heteroaromatic primary amines. Analogues **4**, **5**, **6**, **8**, **9**, **14**, **15**, **16** were prepared by coupling between salicyloyl chloride esters and heteroaromatic primary amines. Analog **3** was prepared by coupling between 2-(methoxycarbonyl)benzoyl chloride and 2-amino-5-nitrothiazole. Analogue **7** was prepared by methylation of nitazoxanide using the methylating agent iodomethane and K_2_CO_3_. Full details are given in the Supporting Information.

### THP-1 differentiation, Mtb co-infection and luciferase assay

THP-1 cells were differentiated for 24 h with 40 µM PMA in RPMI-1640 medium supplemented with 10% FBS and 1% glutamine. For macrophage co-infection, cell suspensions were differentiated in 96-well plates and incubated at 37°C overnight. The cells were washed 3 times with 100 µl RPMI medium before infection. Mtb cultures, grown in Middlebrook 7H9 supplemented with oleic acid-albumin-dextrose-catalase (OADC) and 0.05% Tween 80, were washed with 7H9 medium and the bacteria were opsonized by resuspension in RPMI-1640 medium containing 10% human serum for 30 min at 37°C. Donor-specific serum was used for the infection of PBMC. The opsonized bacteria were resuspended in RPMI-1640 and added to differentiated THP-1 cells or PBMC at a multiplicity of infection (MOI) of 1 or 10 for 3.5 h at 37°C in 5% CO_2_. The cells were washed gently 3 times with RPMI-1640 to remove non-internalized bacteria and then incubated in 100 µl of RPMI-1640 containing 1% glutamate and 10% FBS. After 24 h, the medium was aspirated and replaced with medium containing test compounds. After 24, 48 or 72 h, the medium was aspirated and 100 µl Bright-Glo reagent (Promega TM052) was added. After 10 min incubation, luciferase activity was measured with aTropix TR7171 luminometer (Applied Biosystems).

### Isolation of human PBMC

Peripheral blood was collected from normal human subjects, in accordance with the ethics approval guidelines as stated above. Blood was collected in BD Vacutainer Plus plastic plasma tubes coated with sodium heparin (158 USP units) (BD Diagnostics #367874). Blood from a single donor was diluted 1∶1 with PBS, layered over Ficoll-Paque PLUS (Stemcell Technologies #07957), and centrifuged at 900 g for 30 min according to manufacturer's instructions. White blood cells were isolated from buffy coats after centrifugation, and their viability was assessed by Trypan blue to be >98%. 5×10^6^white blood cells were plated directly in 96-well assay plates in RPMI-1640 supplemented with 5% donor-specific human serum. Human serum was prepared from the plasma portion of the Ficoll gradient, heat-inactivated at 55°C for 30 min, centrifuged at 3,000×g for 20 min and filter sterilized before use. PBMC were allowed to adhere to plates for 20–24 h, after which cells were washed 2–3 times with supplemented RPMI to remove non-monocytic cells. Adherent cells were subsequently infected or treated with drugs as described above.

### Viability assays

The viability of PBMC or differentiated THP-1 cells infected with *M. tuberculosis* in 96-well plates was measured at 24, 48 and 72 h using the (3-[4,5-Dimethylthiazol-2-yl]-2,5-diphenyl-tetrazolium bromide (MTT) assay (M2128, Sigma) as described [83]. For propidium iodide (PI) and nuclear staining assays, 100 µl of pre-warmed RPMI-1640 containing propidium iodide (Sigma-Aldrich P-4170) and Hoechst 33342was added directly to wells at indicated timepoints, to final concentrations of0.25 µg/ml and 0.5 µg/ml, respectively. Plates were incubated at 37°for 30 min, and scanned using the CellomicsArrayscan VTI automated fluorescence imager. The cells were imaged with a 20× objective with the Hoechst and TRITC (Ch2, red) channel, and data was collected from at least 800 cells per well. The target activation algorithm was applied to obtain a nuclear mask in the Hoechst channel and corresponding cytoplasmic mask applied in the TRITC channel to quantitate cell number and PI positive cells, respectively. The untreated wells were identified as reference wells during the scan. PI-positive cells show up as bright fluorescent objects in Ch2, with a higher intensity than live cells. The data output “% responder_AvgIntenCh2” is the percentage of cells with average intensity in cytoplasmic mask Ch2which had higher average intensity than that of untreated cells. Cell viability was calculated as the proportion of cells that were propidium iodide-negative.

### NQO1 enzymatic activity assay

NQO1 activity was measured using a modified version of the Prochaska microtiter plate bioassay, which measures enzymatic activity based on the menadione-mediated reduction of MTT [50], [51].0.25 µg/ml of purified NQO1 enzyme (human DT Diaphorase, Sigma D1315) in dH_2_O, or fresh lysates from MCF-7 cells were used in each reaction in a 96-well plate format. MCF-7 cells were grown overnight in RPMI in 6-well plates, then washed and resuspended in cold PBS. 9×10^6^ cells were collected by scraping, and lysed in cold 0.5×PBS by passing 15 times through a 1 mL syringe with a 26 gauge ½ in needle. Lysis was performed on ice. The lysates were diluted in cold 0.5×PBS to seed an equivalent of 10,000 cells per well. Wells were then treated with the indicated concentrations of drugs diluted in 1×PBS, at room temperature. After 1 h incubation at 37°C, 200 µL of fresh reaction mixture (25 mM Tris HCl pH 7.4, 0.01% Tween-20, 5 µM FAD, 1 mM G6P, 30 µM NADP, 60 U G6PD, 0.67 mg/mlbovine serum albumin, 0.3 mg/mlMTT, and 25 µM menadione) was added to each well at room temperature. Parallel reactions using reaction mixture without menadione were used as controls, and wells containing reaction mixture only were used as nonenzymatic blanks. After 5 min incubation at room temperature, the reaction was arrested with 0.3 mM dicoumarol in 0.5% DMSO and 5 mM potassium phosphate pH 7.4. Plates were scanned at 595 nm at 5 min intervals with a spectrophotometer.

### Accession numbers (UniProtKB/SwissProt)

LC3 (Q9H492); mTOR (P42345); PKB/Akt (P31749); S6K (P23443); TSC2 (Q7TT21); NQO1 (P15559); glutathione-S-transferase P1 (P09211); p62/sequestosome 1 (Q13501); Nrf2 (Q16236).

## Supporting Information

Figure S1 **EGFP-LC3 localization in cells treated with NTZ or TIZ.** MCF-7 cells stably expressing EGFP-LC3 were incubated for 3 h or 24 h with different concentrations of NTZ, TIZ or DMSO. EGFP-LC3 is depicted in green and DNA in blue. Scale bar, 10 µm.
(TIF)

Figure S2 **Additional nitazoxanide analogues.** These analogues were tested for induction of autophagosome accumulation, induction of EGFP-LC3 processing and mTORC1 inhibition and found to be inactive in all three assays.
(TIF)

Figure S3 **Effect of NTZ on Mtb.** Mtb cultures seeded in liquid medium at OD of 0.1 were treated with drugs at indicated concentrations, and growth of the same culture was measured after 48, and 72 h by (A) OD, or (B) luciferase activity.
(TIF)

Figure S4 **Effect of rapamycin on Mtb.** Differentiated THP-1 cells infected with Mtb H37Rv bearing a luciferase-reporting plasmid were treated with various concentrations rapamycin for 24, 48, and 72 h. Intracellular Mtb was measured as luciferase activity.
(TIF)

Supporting Information **Supplemental materials and methods.**
(DOCX)
