# Supplementary figures and images for: Nitazoxanide Stimulates Autophagy and Inhibits mTORC1 Signaling and Intracellular Proliferation of Mycobacterium tuberculosis
Source: PLoS Pathog. 2012 May 10;8(5):e1002691. doi: 10.1371/journal.ppat.1002691 (PMC3349752; doi:10.1371/journal.ppat.1002691)

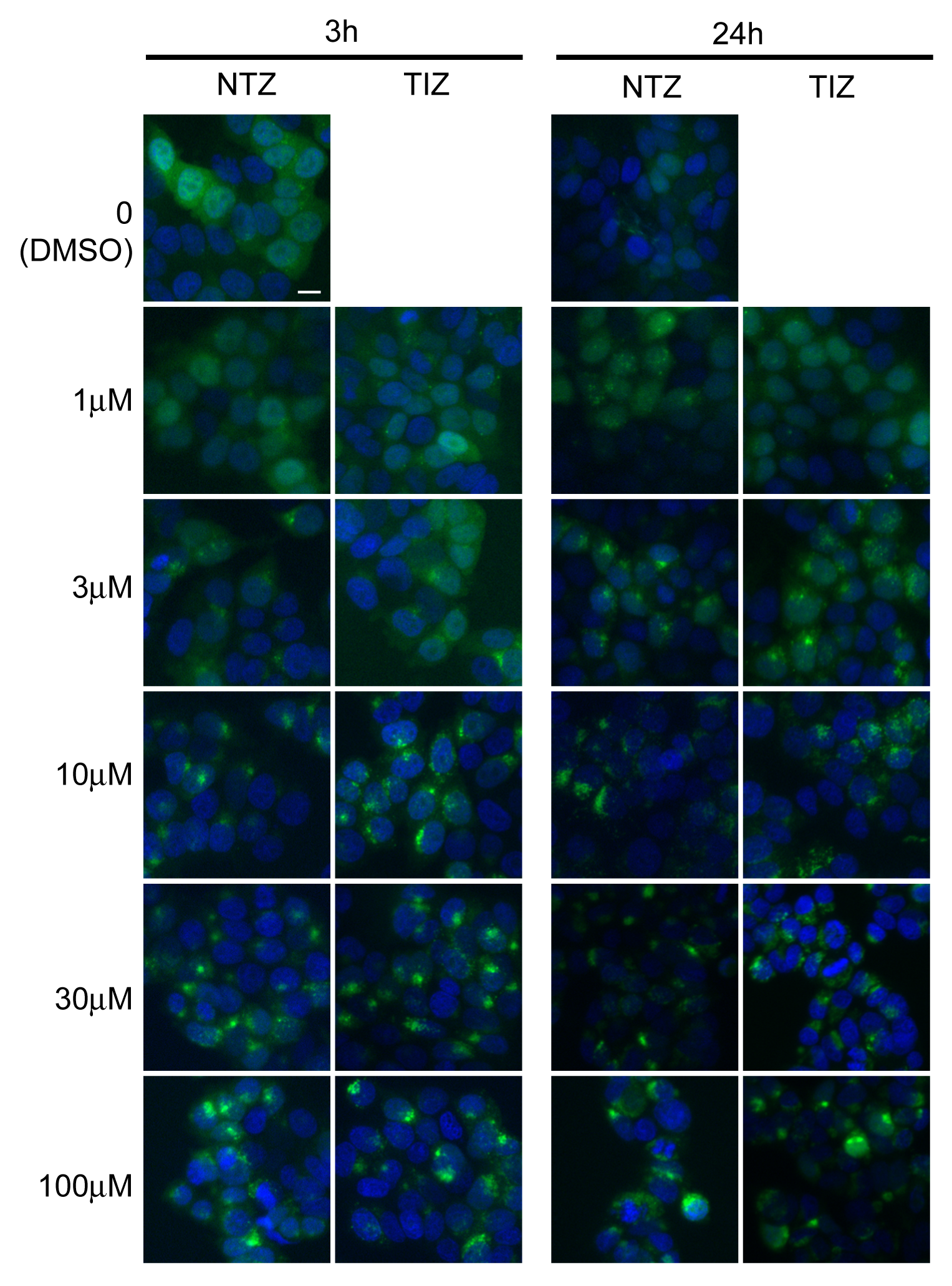

Supplement: Figure S1 — EGFP-LC3 localization in cells treated with NTZ or TIZ. MCF-7 cells stably expressing EGFP-LC3 were incubated for 3 h or 24 h with different concentrations of NTZ, TIZ or DMSO. EGFP-LC3 is depicted in green and DNA in blue. Scale bar, 10 µm. (TIF) [file ppat.1002691.s001.tif]

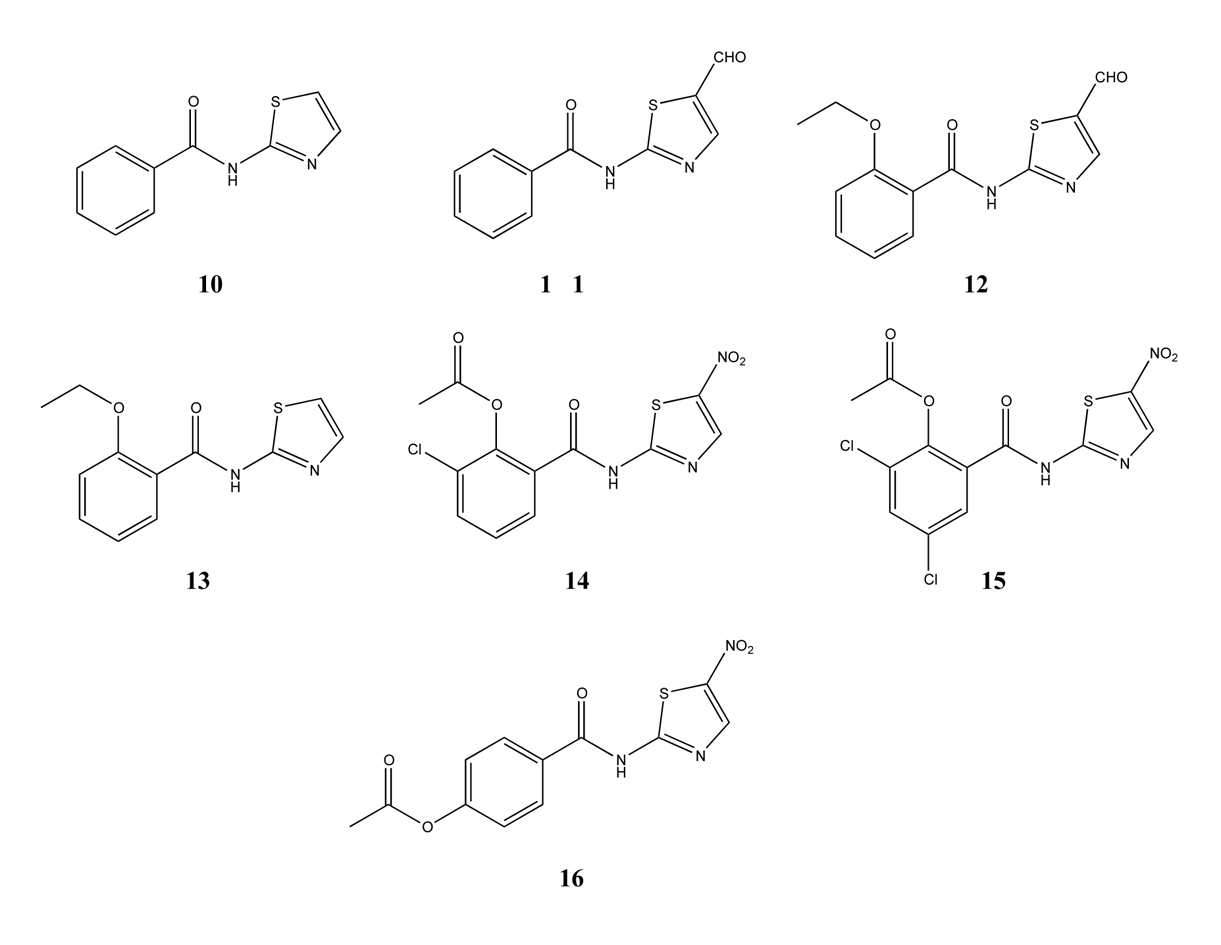

Supplement: Figure S2 — Additional nitazoxanide analogues. These analogues were tested for induction of autophagosome accumulation, induction of EGFP-LC3 processing and mTORC1 inhibition and found to be inactive in all three assays. (TIF) [file ppat.1002691.s002.tif]

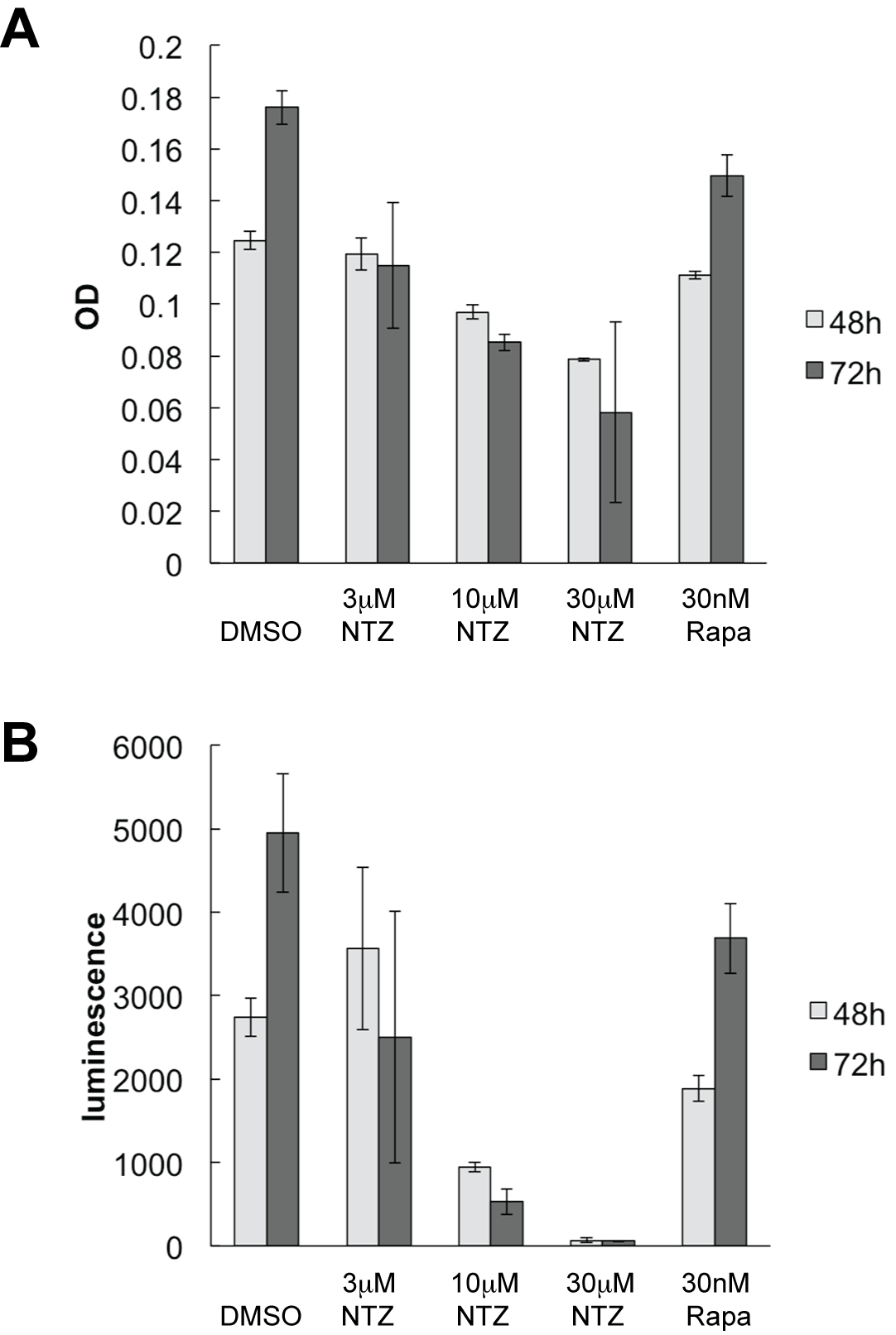

Supplement: Figure S3 — Effect of NTZ on Mtb. Mtb cultures seeded in liquid medium at OD of 0.1 were treated with drugs at indicated concentrations, and growth of the same culture was measured after 48, and 72 h by (A) OD, or (B) luciferase activity. (TIF) [file ppat.1002691.s003.tif]

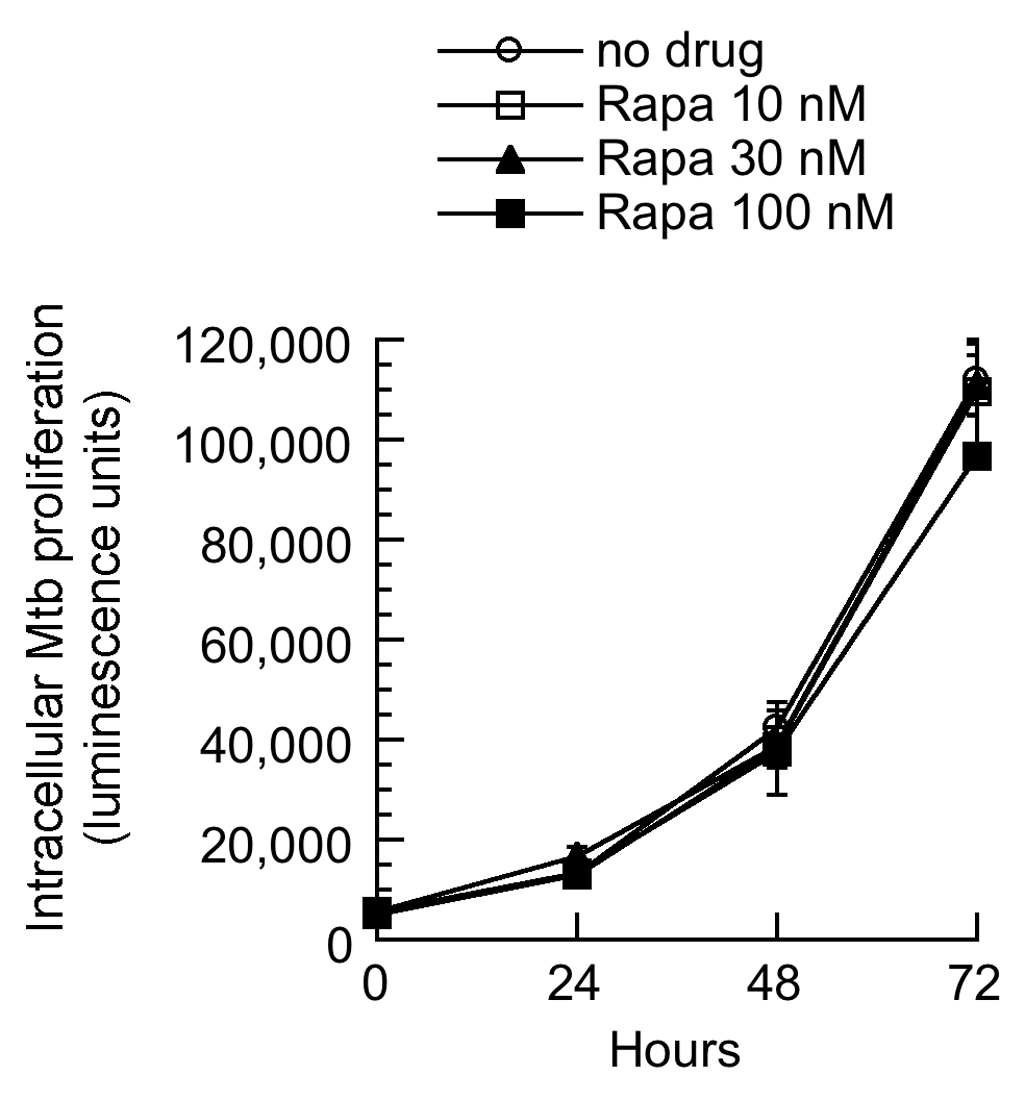

Supplement: Figure S4 — Effect of rapamycin on Mtb. Differentiated THP-1 cells infected with Mtb H37Rv bearing a luciferase-reporting plasmid were treated with various concentrations rapamycin for 24, 48, and 72 h. Intracellular Mtb was measured as luciferase activity. (TIF) [file ppat.1002691.s004.tif]
