# Supplementary material for: Nitazoxanide Stimulates Autophagy and Inhibits mTORC1 Signaling and Intracellular Proliferation of Mycobacterium tuberculosis
Source: PLoS Pathog. 2012 May 10;8(5):e1002691. doi: 10.1371/journal.ppat.1002691 (PMC3349752; doi:10.1371/journal.ppat.1002691)
Supplement: Supporting Information — Supplemental materials and methods. (DOCX) [file ppat.1002691.s005.docx]

**Supporting Information: Materials and Methods**

***In vitro* *M. tuberculosis* proliferation assay**

A starting culture of *M. tuberculosis* H37Rv with OD_600_ of 0.78 was washed once with Middlebrook 7H9 with 0.05% Tween 80, then resuspended in 7H9 supplemented with oleic acid-albumin-dextrose-catalase with 0.05% Tween 80 to final OD_600_ of 0.1. 990 µl of suspension was added to 1.5 ml tubes, and 10 µl of 100X drugs at various concentrations diluted in same growth media was added. Cultures were incubated in a 37°C non-shaking incubator. At each time point (48 and 72 h), cultures were taken for OD_600_ measurements with a spectrophotometer, or luciferase luminescence measurements as described in the main text.

**Synthesis of Nitazoxanide Analogues**

All reactions were carried out with dry solvents under a nitrogen atmosphere in anhydrous conditions, unless otherwise noted. Commercially available anhydrous tetrahydrofuran (THF), dimethylformamide (DMF), and dichloromethane (CH_2_Cl_2_) were used to perform the reactions, unless otherwise stated. Yields refer to chromatographically and spectroscopically (^1^H NMR, ^13^C NMR) homogeneous materials, unless otherwise stated. Reagents were purchased at the highest commercial quality and used without further purification, unless otherwise stated. Reactions were monitored by thin layer chromatography (TLC) carried out on Merck Type 5554 silica gel plates using UV light as visualizing agent and a solution of *p*-anisaldehyde in ethanol/aqueous H_2_SO_4_, and heat as developing agents. Flash chromatography was performed using Silicycle Ultra Pure silica gel (230-400 mesh). The ^1^H and ^13^C NMR spectra were recorded on a Bruker AV-600 spectrometer with a 5 mm CPTCI cryoprobe. ^1^H chemical shifts are referenced to the residual DMSO- *d_6_* signal (*δ* 2.50 ppm) and ^13^C chemical shifts are referenced to the DMSO- *d_6_* solvent peak (*δ* 39.51 ppm). The following abbreviations were used to explain the multiplicities: s = singlet, d = doublet, t = triplet, q = quartet, m = multiplet, quin = quintuplet, sext = sextet, sep = septet, b = broad. Low resolution ESI +/- were recorded on Bruker Esquire LC ion trap mass spectrometer equipped with an electrospray ion source. The solvent for ESI-MS experiments was methanol. The sample solution concentration was 10 *μ*M. It was infused into the ion source by a syringe pump at flow rate of 10 μL/min. High resolution ESI+ were recorded on a Micromass LCT time-of-flight (TOF) mass spectrometer equipped with an electrospray ion source. The samples were dissolved in methanol. The working solutions were 20 *μ*M. Flow rate: 20 *μ*L min-1; sample cone: 90V; source temperature: 120 ºC; desolvation temperature: 120 ºC.

For compounds: **1**, **2**, **8**, **9**, **10**, **11**, **12**, **13**:

**Method A (aromatic acyl chloride formation and coupling with heteroaromatic primary amine).** To a solution of aromatic carboxylic acid (0.001 mol) in dichloromethane (10 mL) was added thionyl chloride (144 *µ*L, 0.002 mol) and a catalytic amount of DMF (50 µL). The solution was stirred for 1 hour. TLC analysis of the reaction mixture shows a complete disappearance of the starting material. The solvent was removed under nitrogen flow to give the aromatic acid chloride as a yellow oil. The aromatic acyl chloride was dissolved in THF (10 mL) and the heteroaromatic primary amine (0.001 mol) was added to the resultant solution. The solution was stirred for 10 minutes then triethylamine (279 *µ*L, 0.002 mol) was added slowly to the mixture. The reaction was stirred for 1 hour. TLC analysis of the reaction mixture shows a complete disappearance of the starting material. The reaction was then quenched with 4 0mL of 10% hydrochloric acid solution. The solution was extracted with dichloromethane (2 x 30 mL). The combined organic phase was washed with a saturated sodium bicarbonate solution (2 x 20 mL), dried and evaporated under vacuum. The resulting residue was purified by flash column chromatography (silica gel, step gradient from 9:1 dichloromethane/MeOH to MeOH) to obtain analytically pure product.

For compounds: **4**, **5**, **6**, **14**, **15**, **16**:

**Method B (synthesis of salicylic acid esters, acyl chloride formation and coupling with heteroaromatic primary amine).** To a solution of salicylic acid (0.001 mol) in THF (10 mL) was added the aliphatic anhydride (0.005 mol) and a catalytic amount of 4-dimethylaminopyridine (12 mg, 0.0001 mol). The solution was stirred for 1 hour. TLC analysis of the reaction mixture shows a complete disappearance of the starting material. The reaction was then quenched with 40 mL of water. The solution was extracted with dichloromethane (2 x 30 mL). The combined organic phase was washed with water (2 x 20 mL), dried and evaporated under vacuum to give salicylic acid ester as a white solid. The salicylic acid ester was dissolved in dichloromethane (10 mL). Then thionyl chloride (144 *µ*L, 0.002 mol) and a catalytic amount of DMF (50 µL) were added to the solution. The reaction was stirred for 1 hour. TLC analysis of the reaction mixture shows a complete disappearance of the starting material. The solvent was removed under nitrogen flow to give the salicylic acyl chloride as a yellow oil. The salicylic acid chloride was dissolved in THF (10 mL) and the heteroaromatic primary amine (0.001 mol) was added to the resultant solution. The solution was stirred for 10 minutes then triethylamine (279 *µ*L, 0.002 mol) was added slowly to the mixture. The reaction was stirred for 1 hour. TLC analysis of the reaction mixture shows a complete disappearance of the starting material. The reaction was then quenched with 40mL of 10% hydrochloric acid solution. The solution was extracted with dichloromethane (2 x 30 mL). The combined organic phase was washed with a saturated sodium bicarbonate solution (2 x 20 mL), dried and evaporated under vacuum. The resulting residue was purified by flash column chromatography (silica gel, step gradient from 9:1 dichloromethane/MeOH to MeOH) to obtain analytically pure product.

***N*-(5-Nitrothiazol-2-yl)benzamide (1)**. Method A yielded compound **1** (142 mg, 57%) as a yellow solid. ^1^H NMR (600 MHz, DMSO-*d*_6_) *δ* 7.59 (t, *J* = 7.8 Hz, 2H), 7.70 (t, *J* = 7.8 Hz, 1H), 8.13 (d, *J* = 7.8 Hz, 2H), 8.73 (s, 1H), 13.61 (bs, 1H). ^13^C NMR (150 MHz, DMSO-*d*_6_) *δ* 128.6, 128.8, 130.9, 133.5, 142.0, 142.7, 162.8, 166.6. HRESIMS [M + H] ^+^ *m/z* 248.0124 (calcd for C_10_H_6_N_3_O_3_S, 248.0130).

**2-Ethoxy-*N*-(5-nitrothiazol-2-yl)benzamide** **(2)**. Method A yielded compound **2** (129 mg, 41%) as a yellow solid. ^1^H NMR (600 MHz, DMSO-*d*_6_) *δ* 1.39 (t, *J* = 7.2 Hz, 3H), 4.20 (q, *J* = 7.2 Hz, 2H), 7.11 (t, *J* = 7.2 Hz, 1H), 7.23 (d, *J* = 8.4 Hz, 1H), 7.60 (dt, *J* = 7.5, 1.8 Hz, 1H), 7.74 (dd, *J* = 7.5, 1.8 Hz, 1H), 8.69 (s, 1H), 12.73 (bs, 1H). ^13^C NMR (150 MHz, DMSO-*d*_6_) *δ* 14.4, 64.5, 113.2, 120.6, 120.7, 130.5, 134.3, 142.0, 142.8, 156.8, 161.4, 165.6. HRESIMS [M + Na]^+^ *m/z* 316.0363 (calcd for C_12_H_11_N_3_O_4_SNa, 316.0368).

**Methyl 2-(5-nitrothiazol-2-ylcarbamoyl)benzoate** **(3)**. To a solution of phthalic anhydride (148 mg, 0.001 mol) in THF (10 mL) was added a catalytic amount of 4-dimethylaminopyridine (12 mg, 0.0001 mol). The solution was stirred for 30 minutes. Then methanol (5 mL) was added to the reaction mixture which was stirred for 1 hour. TLC analysis of the reaction mixture shows a complete disappearance of the starting material. The reaction was then quenched with 40mL of water. The solution was extracted with chloroform (2 x 30 mL). The combined organic phase was washed with water (2 x 20 mL), dried and evaporated under vacuum to give 2-(methoxycarbonyl)benzoic acid as a white solid. 2-(methoxycarbonyl)benzoic acid was dissolved in dichloromethane (10 mL). Then thionyl chloride (144 *µ*L, 0.002 mol) and a catalytic amount of DMF (50 µL) were added to the solution. The reaction was stirred for 1 hour. TLC analysis of the reaction mixture shows a complete disappearance of the starting material. The solvent was removed under nitrogen flow to give 2-(methoxycarbonyl)benzoic acyl chloride as a yellow oil. The 2-(methoxycarbonyl)benzoic acyl chloride was dissolved in THF (10 mL) and 2-amino-5-nitrothiazole (145 mg, 0.001 mol) was added to the resultant solution. The solution was stirred for 10 minutes then triethylamine (279 *µ*L, 0.002 mol) was added to the mixture. The reaction was stirred for 1 hour. TLC analysis of the reaction mixture shows a complete disappearance of the starting material. The reaction was then quenched with 40mL of 10% hydrochloric acid solution. The solution was extracted with dichloromethane (2 x 30 mL). The combined organic phase was washed with a saturated sodium bicarbonate solution (2 x 20 mL), dried and evaporated under vacuum. The resulting residue was purified by column chromatography (silica gel, step gradient from 9:1 dichloromethane/MeOH to MeOH) to afford the pure product **3** (92 mg, 30%) as a yellow solid. ^1^H NMR (600 MHz, DMSO-*d*_6_) *δ* 3.78 (s, 3H), 7.73 (m, 3H), 7.94 (d, *J* = 7.8 Hz, 1H), 8.67 (s, 1H), 13.59 (bs, 1H). ^13^C NMR (150 MHz, DMSO-*d*_6_) *δ* 52.6, 124.0, 128.4, 129.0, 129.5, 131.0, 132.4, 135.2, 135.3, 142.9, 166.1, 168.8. HRESIMS [M + Na]^+^ m/z 330.0164 (calcd for C_12_H_9_N_3_O_5_SNa, 330.0161).

**2-(5-Nitrothiazol-2-ylcarbamoyl)phenyl propionate (4)**. Method B yielded compound **4** (132 mg, 41%) as a yellow solid. ^1^H NMR (600 MHz, DMSO-*d*_6_) *δ* 1.03 (t, *J* = 7.2 Hz, 3H), 2.57 (q, *J* = 7.2 Hz, 2H), 7.32 (d, *J* = 7.8 Hz, 1H), 7.45 (t, *J* = 7.8 Hz, 1H), 7.69 (dt, *J* = 7.8, 1.2 Hz, 1H), 7.84 (dd, *J* = 7.8, 1.2 Hz, 1H), 8.70 (s, 1H), 13.62 (bs, 1H). ^13^C NMR (150 MHz, DMSO-*d*_6_) *δ* 8.8, 27.0, 123.4, 125.6, 125.9, 129.8, 133.5, 142.1, 142.6, 148.7, 161.9, 165.4, 172.2. HRESIMS [M + H]^+^ *m/z* 322.0493 (calcd for C_13_H_12_N_3_O_5_S 322.0498).

**2-(5-Nitrothiazol-2-ylcarbamoyl)phenyl isobutyrate** **(5)**. Method B yielded compound **5** (124 mg, 37%) as a yellow solid. ^1^H NMR (600 MHz, DMSO-*d*_6_) *δ* 1.18 (d, *J* = 6.6 Hz, 6H), 2.79 (sep, *J* = 7.2 Hz, 1H), 7.32 (d, *J* = 7.8 Hz, 1H), 7.45 (t, *J* = 7.8 Hz, 1H), 7.68 (dt, *J* = 7.8, 1.2 Hz, 1H), 7.82 (dd, *J* = 7.8, 1.2 Hz, 1H), 8.70 (s, 1H), 13.62 (bs, 1H). ^13^C NMR (150 MHz, DMSO-*d*_6_) *δ* 18.5, 33.4, 123.3, 125.9, 126.0, 129.7, 133.4, 142.1, 142.6, 148.5, 161.8, 165.4, 174.5. HRESIMS [M + Na]^+^ *m/z* 358.0467 (calcd for C_14_H_13_N_3_O_5_SNa 358.0474).

**2-(5-Nitrothiazol-2-ylcarbamoyl)phenyl pivalate (6)**. Method B yielded compound **6** (119 mg, 34%) as a yellow solid. ^1^H NMR (600 MHz, DMSO-*d*_6_) *δ* 1.25 (s, 9H), 7.29 (d, *J* = 7.8 Hz, 1H), 7.44 (t, *J* = 7.2 Hz, 1H), 7.67 (dt, *J* = 7.2, 1.2 Hz, 1H), 7.81 (dd, *J* = 7.8, 1.2 Hz, 1H), 8.69 (s, 1H), 13.60 (bs, 1H). ^13^C NMR (150 MHz, DMSO-*d*_6_) *δ* 26.7, 38.5, 123.2, 126.0, 126.2, 129.6, 133.2, 142.0, 142.7, 148.6, 162.0, 165.5, 175.9. HRESIMS [M + Na]^+^ *m/z* 372.0623 (calcd for C_15_H_15_N_3_O_5_SNa 372.0630).

**2-(Methyl(5-nitrothiazol-2-yl)carbamoyl)phenyl acetate (7)**. To a solution of acetylsalicylic acid (180 mg, 0.001 mol) in dichloromethane (10 mL) was added thionyl chloride (144 *µ*L, 0.002 mol) and a catalytic amount of DMF (50 µL). The solution was stirred for 1 hour. TLC analysis of the reaction mixture shows a complete disappearance of the starting material. The solvent was removed under nitrogen flow to give acetylsalicylic acyl chloride as a yellow oil. The acetylsalicylic acyl chloride was dissolved in THF (10 mL) and 2-amino-5-nitrothiazole (145 mg, 0.001 mol) was added to the resultant solution. The solution was stirred for 10 minutes then triethylamine (279 *µ*L, 0.002 mol) was added to the mixture. The reaction was stirred for 1 hour. TLC analysis of the reaction mixture shows a complete disappearance of the starting material. The reaction was then quenched with 40mL of 10% hydrochloric acid solution. The solution was extracted with dichloromethane (2 x 30 mL). The combined organic phase was washed with a saturated sodium bicarbonate solution (2 x 20 mL), dried and evaporated under vacuum. The resulting residue was purified by flash column chromatography (silica gel, step gradient from 9:1 dichloromethane/MeOH to MeOH) to obtain pure nitazoxanide. Nitazoxanide was added to a stirred suspension of K_2_CO_3_ (690 mg, 0.005 mol) in DMF (10 mL). The methylating agent iodomethane (62 *µ*L, 0.001 mol) was then added slowly to the reaction mixture. The solution was stirred for 24 hours. TLC analysis of the reaction mixture shows a complete disappearance of the starting material. The reaction was then quenched with 40mL of 10% hydrochloric acid solution. The solution was extracted with dichloromethane (2 x 30 mL). The combined organic phase was washed with water (2 x 20 mL), dried and evaporated under vacuum. The resulting residue was purified by column chromatography (silica gel, step gradient from 9:1 dichloromethane/MeOH to 100% MeOH) to afford the the pure product **7** (177 mg, 55%) as a yellow solid. ^1^H NMR (600 MHz, DMSO-*d*_6_) *δ* 2.32 (s, 3H), 3.83 (s, 3H), 7.22 (d, *J* = 8.4 Hz, 1H), 7.43 (t, *J* = 7.2 Hz, 1H), 7.65 (dt, *J* = 7.8, 1.2 Hz, 1H), 8.30 (dd, *J* = 7.8, 1.2 Hz, 1H), 9.19 (s, 1H). ^13^C NMR (150 MHz, DMSO-*d*_6_) *δ* 21.1, 36.7, 123.9, 126.0, 128.0, 131.6, 133.5, 133.6, 136.1, 150.3, 165.1, 169.2, 172.8. HRESIMS [M + Na]^+^ *m/z* 344.0312 (calcd for C_13_H_11_N_3_O_5_SNa, 344.0317).

**2-(Thiazol-2-ylcarbamoyl)phenyl acetate** **(8)**. Method A yielded compound **8** (131 mg, 50%) as a white solid. ^1^H NMR (600 MHz, DMSO-*d*_6_) *δ* 2.22 (s, 3H), 7.27 (d, *J* = 8.4 Hz, 1H), 7.28 (d, *J* = 3.6 Hz, 1H), 7.40 (t, *J* = 7.8 Hz, 1H), 7.54 (d, *J* = 3.6 Hz, 1H), 7.62 (dt, *J* = 7.8, 1.2 Hz, 1H), 7.77 (d, *J* = 7.8 Hz, 1H), 12.58 (bs, 1H). ^13^C NMR (150 MHz, DMSO-*d*_6_) *δ* 20.7, 113.9, 123.3, 125.9, 126.9, 129.6, 132.6, 137.8, 148.5, 158.0, 163.9, 168.9. HRESIMS [M + Na]^+^ *m/z* 285.0316 (calcd for C_12_H_10_N_2_O_3_SNa, 285.0310).

**2-(5-Formylthiazol-2-ylcarbamoyl)phenyl acetate** **(9)**. Method A yielded the title compound **9** (157 mg, 54%) as a white solid. ^1^H NMR (600 MHz, DMSO-*d*_6_) *δ* 2.23 (s, 3H), 7.30 (d, *J* = 7.8 Hz, 1H), 7.43 (t, *J* = 7.8 Hz, 1H), 7.66 (dt, *J* = 7.2, 1.2 Hz, 1H), 7.82 (d, *J* = 7.2 Hz, 1H), 8.49 (s, 1H), 10.00 (s, 1H), 13.25 (bs, 1H). ^13^C NMR (150 MHz, DMSO-*d*_6_) *δ* 20.7, 117.2, 123.4, 125.9, 126.1, 129.7, 132.4, 133.2, 148.6, 150.5, 164.0, 168.9, 184.3. HRESIMS [M + Na]^+^ *m/z* 313.0261 (calcd for C_13_H_10_N_2_O_4_SNa 313.0259).

***N*-(Thiazol-2-yl)benzamide** **(10)**. Method A yielded compound **10** (139 mg, 68%) as a white solid. ^1^H NMR (600 MHz, DMSO-*d*_6_) *δ* 7.29 (d, *J* = 3.6 Hz, 1H), 7.55 (t, *J* = 7.8 Hz, 2H), 7.57 (d, *J* = 3.6 Hz, 1H), 7.63 (t, *J* = 7.2 Hz, 1H), 8.09 (d, *J* = 7.2 Hz, 2H), 12.64 (bs, 1H). ^13^C NMR (150 MHz, DMSO-*d*_6_) *δ* 113.9, 128.1, 128.6, 132.2, 132.6, 137.8, 158.7, 165.1. HRESIMS [M + Na]^+^ *m/z* 227.0259 (calcd for C_10_H_8_N_2_OSNa, 227.0255).

***N*-(5-Formylthiazol-2-yl)benzamide (11)**. Method A yielded compound **11** (142 mg, 61%) as a white solid. ^1^H NMR (600 MHz, DMSO-*d*_6_) *δ* 7.58 (t, *J* = 7.8 Hz, 2H), 7.68 (t, *J* = 7.8 Hz, 1H), 8.12 (d, *J* = 7.2 Hz, 2H), 8.51 (s, 1H), 10.00 (s, 1H), 13.26 (bs, 1H). ^13^C NMR (150 MHz, DMSO-*d*_6_) *δ* 128.5, 128.7, 131.4, 132.3, 133.2, 150.6, 164.8, 166.0, 184.3. HRESIMS [M + H]^+^ *m/z* 233.0387 (calcd for C_11_H_9_N_2_O_2_S, 233.0385).

**2-Ethoxy-*N*-(5-formylthiazol-2-yl)benzamide (12)**. Method A yielded compound **12** (160 mg, 58%) as a white solid. ^1^H NMR (600 MHz, DMSO-*d*_6_) *δ* 1.41 (t, *J* = 7.2 Hz, 3H), 4.22 (q, *J* = 7.2 Hz, 2H), 7.11 (t, *J* = 7.8 Hz, 1H), 7.23 (d, *J* = 8.4 Hz, 1H), 7.59 (dt, *J* = 7.8, 1.8 Hz, 1H), 7.77 (dd, *J* = 7.5, 1.8 Hz, 1H), 8.48 (s, 1H), 10.00 (s, 1H), 12.37 (bs, 1H). ^13^C NMR (150 MHz, DMSO-*d*_6_) *δ* 14.5, 64.6, 113.3, 120.8, 120.9, 130.5, 132.4, 134.1, 150.8, 156.8, 163.5, 164.8, 184.3. HRESIMS [M + Na]^+^ *m/z* 299.0473 (calcd for C_13_H_12_N_2_O_3_SNa, 299.0466).

**2-Ethoxy-*N*-(thiazol-2-yl)benzamide** **(13)**. Method A yielded compound **13** (149 mg, 60%) as a white solid. ^1^H NMR (600 MHz, DMSO-*d*_6_) *δ* 1.43 (t, *J* = 6.6 Hz, 3H), 4.24 (q, *J* = 6.6 Hz, 2H), 7.11 (t, *J* = 7.5 Hz, 1H), 7.22 (d, *J* = 8.4 Hz, 1H), 7.29 (d, *J* = 3.6 Hz, 1H), 7.53 (d, *J* = 3.6 Hz, 1H), 7.57 (dt, *J* = 7.5, 1.8 Hz, 1H), 7.82 (dd, *J* = 7.8, 1.8 Hz, 1H), 11.78 (s, 1H). ^13^C NMR (150 MHz, DMSO-*d*_6_) *δ* 14.5, 64.7, 113.3, 114.0, 120.8, 120.9, 130.7, 133.7, 138.0, 156.6, 157.5, 163.3. HRESIMS [M + Na]^+^ *m/z* 271.0515 (calcd for C_12_H_12_N_2_O_2_SNa, 271.0517).

**2-Chloro-6-(5-nitrothiazol-2-ylcarbamoyl)phenyl acetate (14)**. Method B yielded compound **14** (109 mg, 32%) as a yellow solid. ^1^H NMR (600 MHz, DMSO-*d*_6_) *δ* 2.32 (s, 3H), 7.50 (t, *J* = 7.8 Hz, 1H), 7.84 (dd, *J* = 7.8, 1.2 Hz, 1H), 7.87 (dd, *J* = 8.4, 1.2 Hz, 1H), 8.72 (s, 1H), 13.76 (bs, 1H).^13^C NMR (150 MHz, DMSO-*d*_6_) *δ* 20.2, 127.3, 127.5, 127.8, 128.8, 133.7, 142.2, 142.5, 144.9, 161.9, 164.6, 167.9. HRESIMS [M + Na]^+^ *m/z* 363.9767 (calcd for C_12_H_8_ClN_3_O_5_SNa, 363.9771).

**2,4-Dichloro-6-(5-nitrothiazol-2-ylcarbamoyl)phenyl acetate (15)**. Method B yielded compound **15** (112 mg, 30%) as a yellow solid. ^1^H NMR (600 MHz, DMSO-*d*_6_) *δ* 2.33 (s, 3H), 8.00 (d, *J* = 2.4 Hz, 1H), 8.10 (d, *J* = 2.4 Hz, 1H), 8.72 (s, 1H), 13.80 (bs, 1H). ^13^C NMR (150 MHz, DMSO-*d*_6_) *δ* 20.2, 128.7, 128.9, 128.9, 130.7, 132.9, 142.2, 142.5, 144.0, 162.0, 163.6, 167.8. HRESIMS [M + Na]^+^ *m/z* 397.9389 (calcd for C_12_H_7_Cl_2_N_3_O_5_SNa, 397.9381).
